# Label-free metabolic and structural profiling of dynamic biological samples using multimodal optical microscopy with sensorless adaptive optics

**DOI:** 10.1038/s41598-022-06926-w

**Published:** 2022-03-02

**Authors:** Rishyashring R. Iyer, Janet E. Sorrells, Lingxiao Yang, Eric J. Chaney, Darold R. Spillman, Brian E. Tibble, Carlos A. Renteria, Haohua Tu, Mantas Žurauskas, Marina Marjanovic, Stephen A. Boppart

**Affiliations:** 1 Beckman Institute for Advanced Science and Technology, University of Illinois at Urbana-Champaign, Urbana, USA; 2 Department of Electrical and Computer Engineering, University of Illinois at Urbana-Champaign, Urbana, USA; 3 Department of Bioengineering, University of Illinois at Urbana-Champaign, Urbana, USA; 4 The School of Molecular and Cellular Biology, University of Illinois at Urbana-Champaign, Urbana, USA; 5 Carle Illinois College of Medicine, University of Illinois at Urbana-Champaign, Urbana, USA; 6 Cancer Center at Illinois, University of Illinois at Urbana-Champaign, Urbana, USA

**Keywords:** Biomedical engineering, Adaptive optics, Imaging and sensing, Interference microscopy, Multiphoton microscopy, Optical imaging

## Abstract

Label-free optical microscopy has matured as a noninvasive tool for biological imaging; yet, it is criticized for its lack of specificity, slow acquisition and processing times, and weak and noisy optical signals that lead to inaccuracies in quantification. We introduce FOCALS (Fast Optical Coherence, Autofluorescence Lifetime imaging, and Second harmonic generation) microscopy capable of generating NAD(P)H fluorescence lifetime, second harmonic generation (SHG), and polarization-sensitive optical coherence microscopy (OCM) images simultaneously. Multimodal imaging generates quantitative metabolic and morphological profiles of biological samples in vitro, ex vivo, and in vivo. Fast analog detection of fluorescence lifetime and real-time processing on a graphical processing unit enables longitudinal imaging of biological dynamics. We detail the effect of optical aberrations on the accuracy of FLIM beyond the context of undistorting image features. To compensate for the sample-induced aberrations, we implemented a closed-loop single-shot sensorless adaptive optics solution, which uses computational adaptive optics of OCM for wavefront estimation within 2 s and improves the quality of quantitative fluorescence imaging in thick tissues. Multimodal imaging with complementary contrasts improves the specificity and enables multidimensional quantification of the optical signatures in vitro, ex vivo, and in vivo, fast acquisition and real-time processing improve imaging speed by 4–40 × while maintaining enough signal for quantitative nonlinear microscopy, and adaptive optics improves the overall versatility, which enable FOCALS microscopy to overcome the limits of traditional label-free imaging techniques.

## Introduction

Holistic label-free imaging of “life in action” at a cellular level requires fast multimodal profiling of biological samples in their native environment. However, label-free optical imaging techniques lack the specificity of targeted markers and exogenous contrast agents^1^ and have lower quantum efficiency. Translating the optical property measured by the microscope into biologically relevant characteristics is key to the utility of label-free microscopes in biomedicine^2,3^. Measurement of optical properties such as refractive index, birefringence, non-centrosymmetry, and autofluorescence inform physiological processes like diffusion dynamics^4^, extracellular matrix structures^5,6^, or metabolism^7,8^.

Advances in light sources and sensitive detectors have pushed label-free multiphoton microscopy (MPM) to the forefront of emerging imaging technologies^1,7,9–11^. While fluorescent contrasts and probes have certainly made microscopy more colorful and ubiquitous, a variety of intrinsic photophysical processes have been exploited for functional nonlinear microscopy and molecular imaging. Among these, second-harmonic generation (SHG) microscopy is commonly used to image collagen in the tissue microenvironment and cytoskeletal structures to resolve individual fiber strands with sub-micron resolution^5,6^. Similarly, the autofluorescence from the reduced form of nicotinamide adenine dinucleotide (phosphate) (NAD(P)H) can be exploited to derive quantitative metrics like the fluorescence lifetime or redox ratios that describe the cell or tissue metabolic profile. Free and protein-bound NAD(P)H have dramatically different two-photon absorption quantum efficiencies (10–100 ×) and fluorescence lifetimes (300–800 ps for free and 1500–4500 ps for bound)^12,13^. These metrics are typically estimated using fluorescence lifetime imaging microscopy (FLIM). However, autofluorophores like NAD(P)H have lower quantum efficiency compared to exogenous contrast agents^12^. Therefore, longer exposure times must be used to obtain enough photons for good image quality making the microscopy setups slower and more susceptible to external factors of noise such as dark counts, motion artifacts, and sample dynamics. Designs to improve label-free MPM imaging speed often achieve that at the cost of the collected signal intensity^11,13,14^. At these low signal levels, every photon matters; any loss to the fluorescence intensity due to loss during detection or caused by the sample can slow down image acquisition or reduce the accuracy of the estimated metabolic profiles. In an attempt to maximize speed, fast label-free MPM setups are operated at or below the limit for quantification and may require averaging several frames or pixels for extracting quantitative fluorescence parameters^9,11,15–17^.

Typical setups for FLIM of NAD(P)H are slow because FLIM relies on collecting at least 100 fluorescent photons to make an accurate estimate of the fluorescence lifetime^14^, and NAD(P)H is a weak fluorophore that frequently needs long exposure and collection times^12,13^. The detectors used in the prevalent time-correlated single photon counting (TCSPC) FLIM setups also have long dead times, further decreasing the acquisition speed^18^. Advances in photon detection, analog sampling, signal correlators, and laser sources have been exploited to boost the speed of FLIM^18^ to video-rate imaging of bright fluorophores. However, few have been used for measuring NAD(P)H dynamics or the dynamics of similarly weak autofluorescent biomolecules. Indeed, our group was among the first to demonstrate NAD(P)H two-photon FLIM on the order of 1 Hz through the direct analog sampling of the photodetectors at gigahertz rates^11^. We recently demonstrated our fast two-photon FLIM setup capable of generating real-time images of bright fluorophore dynamics at 25 Hz and NAD(P)H FLIM dynamics at 0.6 Hz by creating a graphical-processing-unit (GPU)-based data handling pipeline^15^. A fundamental limitation to the speed of FLIM is the stringent photon budgets required for the technique. Studies have also shown that the precision and accuracy of fluorescence lifetime measurements are drastically affected by the total number of photons detected^19–21^. Additionally, numerous studies have reported inaccuracies in FLIM measurements for weak signals due to the statistical bias for earlier arriving photons in FLIM detectors^22–25^. Similarly, subtle changes to the spatiotemporal coherence of the excitation beam for multiphoton absorption affect the consequent biomolecular dynamics^26^. One of the major reasons for the distortion of images from optical microscopy and attenuation of the signal intensity is optical aberrations, specifically those that are sample induced and can therefore vary during imaging.

Optical aberrations (OA) have thwarted the dream of many a researcher to image diverse and dynamic biological samples. Researchers have been mathematically describing the image distortions caused by OAs at least since 1667^27^. The advent of new imaging modalities and multimodal microscopes demands renewed attention for examining the role of OAs in label-free, dynamic, functional, and quantitative imaging techniques. Adaptive optics (AO) techniques are used to correct the wavefront deformations due to OAs. Early applications of AO for biological imaging were in ophthalmology where the OAs induced by the ocular geometry distort the images of the deeper retinal layers^28–32^. The effect of OAs has been readily studied in optical coherence tomography (OCT) and optical coherence microscopy (OCM) due to their ability to generate the complex-valued scattered field. Coupled with the prevalence of OCT for retinal imaging, several AO solutions have been devised to correct OAs in OCT/ OCM^33–36^. These solutions could either be hardware-based (HAO), which uses a deformable optical surface to modify the optical wavefront, or computational (CAO), where the wavefront is modulated digitally in the spatial frequency domain^36,37^. CAO has not only been utilized for aberration correction^33,38,39^ but also as a new contrast mechanism^40^ and for wavefront sensing^41^. Besides distortions, OAs also drastically reduce the signal intensity at the focal plane^42–45^. These OA effects are compounded in MPM because of the low probability and spatiotemporal requirements of multiphoton absorption^26,46^. Even for bright fluorophores, OAs limit MPM imaging to a few shallow depths in scattering tissues.

Label-free MPM for quantitative fluorescence imaging is more adversely affected by OAs, since endogenous fluorophores and contrasts are up to two orders-of-magnitude weaker than exogenous ones^12^. For FLIM of NAD(P)H, any OA-induced loss to the collected fluorescence signal would lead to a lower number of detected photons which would decrease the accuracy of the estimated metabolic profiles in order to maintain the same imaging speed. Exploring the effects of OAs on FLIM using TCSPC-based techniques is challenging because of the long exposure times involved during which the effects of sample dynamics could dominate over the effects of OAs. In fast FLIM, it is critical to investigate and recognize the effect of OAs and the right AO technique to correct these aberrations without sacrificing speed, versatility, or accuracy. Wavefront correction is only one-half of an AO solution; the optimal correction pattern must be determined first. Previous AO solutions on FLIM and MPM systems have employed both dedicated wavefront sensors and sensorless algorithms for aberration correction that rely on acquiring a series of images at different states of the deformable element to find the optimal compensation pattern^47–49^. Both techniques have disadvantages for dynamic biological imaging. The performance of wavefront sensors in 3D scattering samples is poor. Typical sensorless AO techniques require the acquisition of multiple images that take time and create higher photon exposure^50^. Especially in fluorescence imaging of live samples, these results could be affected by photobleaching, photodamage, or sample motion. Sensorless AO is particularly pertinent to CAO for OCT and OCM, typically implemented during post-processing to improve the image quality. CAO modifies the wavefront using phase masks in the Fourier plane of the complex-valued OCM images. Besides showing how CAO and HAO can individually correct OAs for OCT in ophthalmology and cancer imaging, we had previously implemented a closed-loop solution for sensorless AO, called *AutoAO*, using CAO as the wavefront sensor to find the optimal HAO correction pattern in real-time to extend imaging depths^51^.

**Figure 1:**
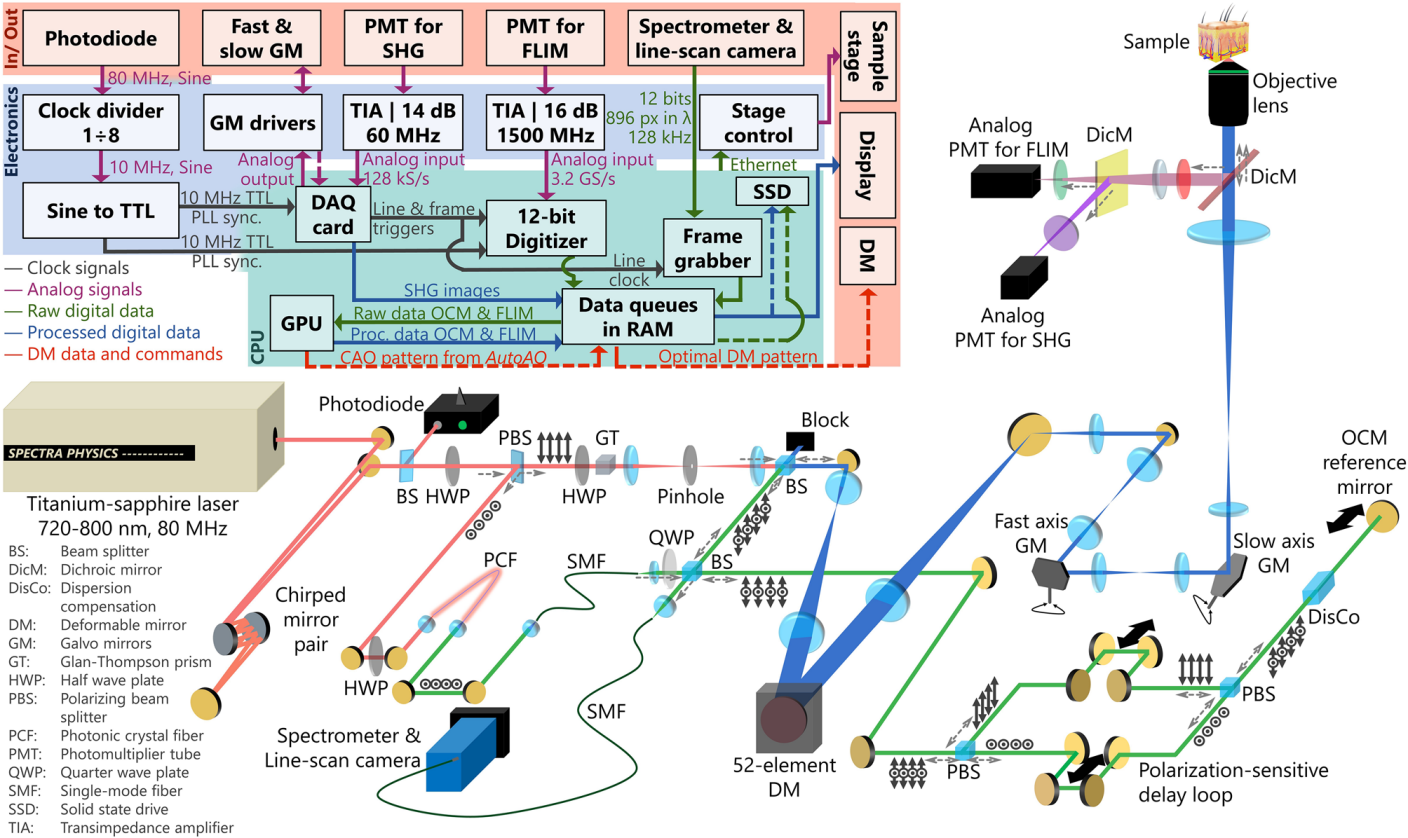
Optical and electronic setup diagram of FOCALS microscopy. In the diagram, the red-colored optical beam corresponds to the FLIM and SHG excitation source (760 ± 5 nm), the green-colored optical beam corresponds to the OCM excitation source (760 ± 80 nm), and the blue-colored optical beam corresponds to the combined OCM-FLIM (and SHG) excitation beams. ↕ and ⊙ correspond to vertical and horizontal polarization states, respectively, and the dashed lines near the beam-spillters indicate the direction of beam propagation (that are pertinent to the excitation and detection paths of FOCALS microscopy).

In this paper, we utilize a novel multimodal setup for label-free 3D dynamic optical imaging that uses a single excitation source, simultaneous detection, real-time processing, and sensorless adaptive optics (Fig. 1). We designed FOCALS (Fast Optical Coherence, Autofluorescent Lifetime imaging, and Second harmonic generation) microscopy for a multimodal approach to overcome the limitations of traditional label-free optical imaging setups. FOCALS microscopy images the complex-valued polarization-sensitive (PS) scattered field, non-centrosymmetry, and the metabolic state of the sample by combining and performing PS-OCM, SHG microscopy, and two-photon FLIM of NAD(P)H, respectively, with a single excitation source. Multimodal imaging that obtains five complementary endogenous contrasts, namely, NAD(P)H fluorescence intensity, NAD(P)H fluorescence lifetime, collagen content, scattering, and birefringence, yields multidimensional characterization of biological samples that are more specific than these contrasts individually. Polarization multiplexing of the OCM reference, analog detection, and gigahertz sampling of the emitted fluorescence with real-time GPU-based processing enables simultaneous fast two-photon FLIM of NAD(P)H, PS-OCM, and SHG microscopy at 0.5 frames per second. We also showed that FOCALS microscopy is 4–40 times faster than comparable techniques for FLIM of NAD(P)H^52–56^ and demonstrated that FOCALS microscopy can generate a holistic label-free representation of the dynamic and diverse tissue microenvironments in 3D and real-time by imaging in vitro, ex vivo, and in vivo. Thus, FOCALS microscopy is uniquely qualified to explore the effects of OAs on FLIM with its capability to image at high speeds and by harnessing prior knowledge of OAs in the context of OCT and OCM. A deformable mirror (DM) placed in the beam path shared by both OCM and MPM beams enables HAO. Having first established the role of OAs in FLIM, a single-shot closed-loop AO solution using the *AutoAO* algorithm was used to improve the accuracy of fluorescence lifetime estimations under low signal conditions, where the coherence-gated imaging channel works both as a wavefront sensor and as a structural imaging channel with scattering contrast. Therefore, even under low signal conditions induced by optical aberrations, FOCALS microscopy can obtain more accurate quantitative fluorescence characteristics using AO, and overcomes the challenges of specificity, speed, and versatility of traditional label-free microscopy setups.

## Results

### FOCALS microscopy generates multimodal images of dynamic and 3D biological samples in real-time

**Figure 2:**
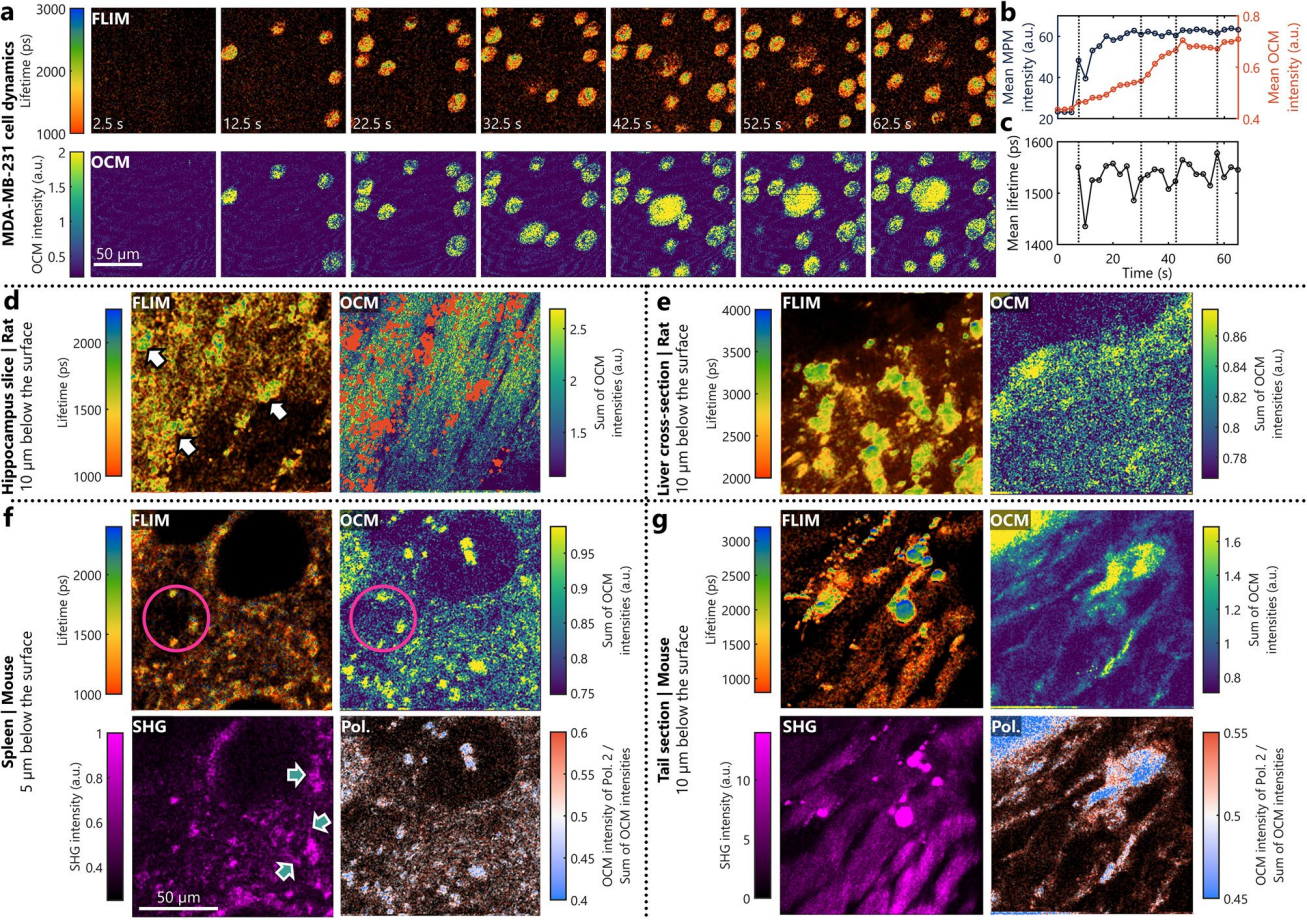
In vitro and ex vivo imaging with FOCALS microscopy. (**a**) Filmstrip of FLIM and OCM images of MDA-MB-231 cells dropped onto a glass surface with media, and acquired at a rate of 0.4 frames per second. Every 5th frame is shown for visualization. (**b**–**c**) Temporal trace of the mean (**b**) NAD(P)H fluorescence (blue) and OCM intensity (orange), and (**c**) lifetime across each frame. The dashed black lines in (**b**,**c**) correspond to inflection points in the OCM intensity trace to match them to changes to the fluorescence intensity and lifetime. (**d**–**e**) FLIM and OCM images of a rat (**d**) hippocampal brain slice and (**e**) liver cross-section 10 µm below the surface. The white arrows indicate the large cell-like structures with a fluorescence lifetime of 1500 ps or longer in the hippocampal slice. The orange dots in the OCM images of the hippocampus indicate pixels whose lifetime is 1500 ps or longer. (**f**) FOCALS microscopy images of a mouse spleen 5 µm below the surface. The pink circle helps highlight the similarities between the FLIM and OCM images. The green arrows indicate subtle fibrous features that appear on SHG and not on any other channels. (**g**) FOCALS microscopy images of a mouse tail section 10 µm below the surface. (Pol.: Polarization ratio expressed as the ratio between the intensity of polarization state 2 and the sum of the two OCM intensity states).

The features of FOCALS microscopy are highlighted by imaging the dynamics of in vitro cells and multimodal profiling of various tissue samples both ex vivo and in vivo. First, we demonstrate the speed and multimodality of FOCALS microscopy by observing the dynamics of cancer cells adhering to a flat glass surface. Cell plating is a routine process for establishing cell cultures that researchers seldom give a second thought about; yet, observing the structural and metabolic profiles of this dynamic process could yield insight into the cells’ interaction with the substrate and the metabolic changes that cells undergo in the in vitro model. The images in the filmstrip shown in Fig. 2a (and Supplementary Fig. 1a) were acquired at 0.4 Hz and processed on a GPU in real-time. The focal plane was 2 µm above the glass surface to avoid the bright background from the glass surface in the en face OCM images. The mean intensities of both OCM and NAD(P)H fluorescence and the NAD(P)H fluorescence lifetimes are plotted to analyze dynamics quantitatively (Fig. 2b–c). Four inflection points were identified where new clusters of cells arrived within the en face planes of interest in OCM (highlighted with black dotted lines in Fig. 2b–c), corresponding to the increase in the fluorescence intensity. At each of these instances, a batch of cells appears within the plane of interest and the OCM intensity is linearly increasing. At each of these points, there is an associated sudden initial decrease to the mean fluorescence lifetime, which increases gradually after the cells land on the glass surface (See Supplementary Fig. 1b–d for the median intensity, lifetime, and phasors and their respective deviations). Bound NAD(P)H has a longer fluorescence lifetime compared to free NAD(P)H and implies increased mitochondrial metabolic activity. These results suggest that the cells in suspension were in a different metabolic state (with lower NAD(P)H lifetime) than the cells adherent to the glass surface, which had higher NAD(P)H fluorescence lifetime. Using FOCALS microscopy, their structural and metabolic profiles were observed in real-time (Supplementary Movie 1).

Second, we demonstrate the versatility of FOCALS microscopy by imaging various freshly extracted tissues from healthy mice and rats. Figure 2d–g shows the multimodal images of hippocampus and liver tissue cross-sections from rats as well as the spleen and tail from a mouse. For this demonstration, we chose to image close to the surface of the tissue samples. In Fig. 2d, the regions indicated by the white arrows in the FLIM images correspond to the cell-like structures in the rat hippocampus that are spread amidst clusters of smaller features of varying lifetimes. The structural context for this spread is apparent in the OCM images, where the points with a fluorescence lifetime of 1500 ps or greater (indicated by the orange dots) populate the gaps in the fiber structures. The resolution of OCM is sufficient to resolve individual fibers, providing structural context for the metabolic profile obtained using FLIM. Supplementary Fig. 2 shows raw OCM images revealing both the fibers and the scattering structures that indicate that the structures highlighted by the white arrows in Fig. 2d in the FLIM images are, like cells, more weakly scattering than the surrounding fibers. Similarly, the image from the liver cross-section has several distinctly bright cellular structures with lifetimes longer than 3000 ps (Fig. 2e). However, the corresponding OCM image is very uniform, where these structures with longer lifetimes are not visually distinct from the extracellular matrix. The structural similarities between the different modalities are more apparent in the spleen images (Fig. 2f), where the structures in OCM with high intensity are similar to the features highlighted by the pink circle in the FLIM images. Examination of the PS-OCM images reveals that these features have nearly equal intensities for both polarization states (white), unlike the extracellular matrix, which has a distinct bias towards polarization state 1 (red), confirming the presence of birefringence. Since the SHG signal was extremely weak, the autofluorescence leaking into the SHG PMT comprises a majority of the features in the SHG image of the spleen. Nonetheless, the green arrows in the SHG images highlight the few subtle structures found in the sample that are different from the structures in the FLIM images, corresponding to the observed birefringence. Finally, the multimodal images of the tail show a similar correlation between the individual modalities (Fig. 2g), where collagen-rich structures in the tail cause a strong SHG signal, have relatively scattering boundaries in OCM, and a strong bias in polarization. While some of the fluorescent signals with high intensity and fluorescence lifetime of 2000 ps or greater leak into the SHG channel and the weak fluorescence of collagen is apparent in the NAD(P)H measurements with a low fluorescence lifetime, the relative intensities in the two channels and FLIM can help specify the origin of these signals more accurately than the individual modality.

**Figure 3:**
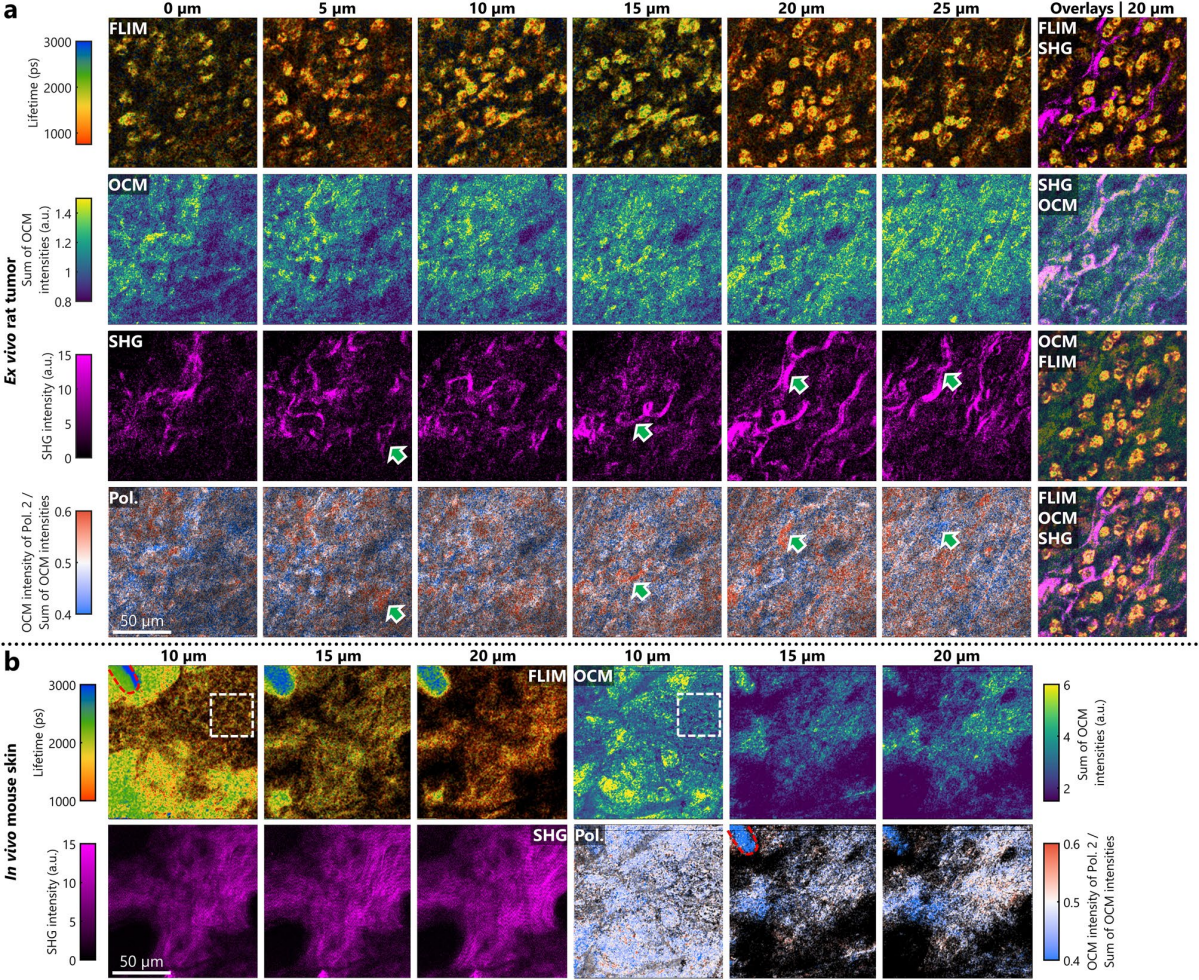
3D FOCALS microscopy images of an ex vivo rat tumor and in vivo mouse skin. (**a**) FOCALS microscopy images of an ex vivo rat mammary tumor at 6 different depths over 25 µm. The green arrows indicate regions where the fibers that appear in SHG affect the polarization ratios in the PS-OCM image. Different overlay combinations of the modalities in FOCALS microscopy highlight the complementary nature of each modality in visualizing the tumor microenvironment. (**b**) FOCALS microscopy images of in vivo mouse skin at 3 different depths over 15 µm. The region in the white dashed box highlights the cells in the stratum granulosum and the red dashed oval region in the top left corner of the frame highlights a bright feature on FLIM that also appears to be strongly birefringent.

The 3D multimodal images of a tumor microenvironment ex vivo were taken one week after injection of rat mammary tumor cells under the skin of a healthy rat (Fig. 3a). The metabolic profile associated with the state of NAD(P)H of individual cells in the environment at each depth is apparent in the FLIM images. The OCM images provide the spatial context for these cells in the extracellular matrix. The appearance of a relatively thick and chaotic distribution of the collagen fibers in the matrix with the cancer cells dispersed within, seen in the SHG images, is a characteristic of the tumor microenvironment^57–59^. Similar collagen signatures were observed previously in tumor models with undefined boundaries^60,61^. In PS-OCM, these locations also show a trend towards a particular polarization state (indicated by the green arrows in Fig. 3a). For better visualization, we also created overlays of the multimodal images, where the relative locations of cells and the collagen matrix are apparent.

Figure 3b shows the 3D FOCALS microscopy images of mouse skin in vivo. The fluorescence lifetime of each cell in the stratum granulosum could be observed in the regions highlighted in white. Additionally, the distinctly high fluorescence lifetime of the features with high fluorescence intensity, highlighted in red (> 3000 ps) can be correlated with the bias towards a specific polarization state observed in the images showing the ratio of OCM intensities. This could indicate the presence of a hair follicle in the field-of-view based on similar features observed in FLIM studies of skin^62^, characterization of the broad range of fluorescence lifetime values of hair^63^, and strong birefringence of hair follicles observed using PS-OCT^64^. The collagen network in these layers is apparent in the SHG images as well. Interestingly, with its high speed, there are no breathing artifacts observed within a frame acquired using FOCALS microscopy. These results highlight the capability of FOCALS microscopy to generate 3D multimodal images of tissues both ex vivo and in vivo, and in real-time.

### *AutoAO* improves the functional accuracy of FOCALS microscopy

**Figure 4:**
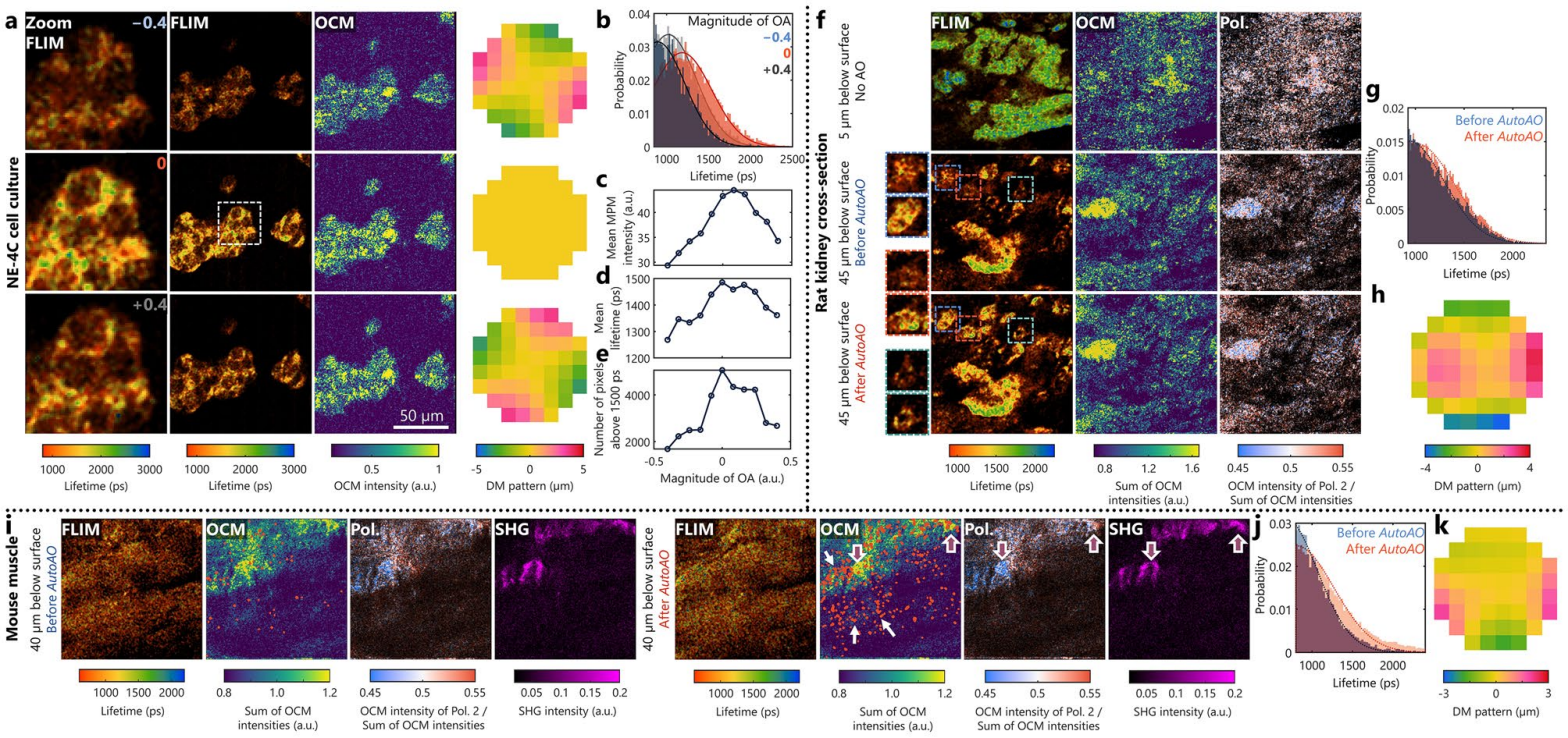
*AutoAO* and FOCALS. (**a**) FLIM and OCM images of NE-4C neuroepithelial cells under different magnitudes of OAs applied to the DM. The first column shows the zoomed-in lifetime images corresponding to the white dashed box in the second column; the third and fourth columns show the OCM images and the DM patterns, respectively. (**b**) Histogram of the NAD(P)H fluorescence lifetime for OA magnitudes −0.4 (blue), 0.4 (gray), and 0 (orange). (**c**–**e**) Plot of (**c**) mean NAD(P)H fluorescence intensity per frame, (**d**) mean lifetime (in ps), and (**e**) number of pixels with a lifetime of 1500 ps or longer against OA magnitude. (**f**) FLIM, OCM, and PS-OCM (polarization ratio) images of a rat kidney 5 µm below the surface, acquired with a flat pattern on the DM, and 45 µm below the surface before and after aberration correction with AO, with three different zoomed-in regions of interest highlighted by the blue, orange, and cyan boxes. (**g**) Histogram of the NAD(P)H fluorescence lifetime for the kidney 45 µm below the surface before (blue) and after (orange) OA correction using *AutoAO*. (**h**) The DM pattern generated by the *AutoAO* algorithm. (**i**) FLIM, OCM, polarization ratio, and SHG images before and after *AutoAO* correction of a mouse muscle 40 µm below the surface. The orange dots correspond to pixels that have a lifetime of 1500 ps or longer. The white arrows indicate the scattering regions in the OCM image around which the orange dots are clustered. The magenta arrows highlight the regions where the fibrous structures are apparent in the SHG images and the polarization ratio is biased towards a particular state. (**j**) Histogram of the NAD(P)H fluorescence lifetime before (blue) and after (orange) OA correction using *AutoAO*. (**k**) The DM pattern generated by the *AutoAO* algorithm.

We explored the effect of the magnitude of OAs on the estimation of fluorescence lifetime of NAD(P)H from cells cultured on a flat surface. Figure 4a presents a series of images of neuroepithelial NE-4C cells for different magnitudes of astigmatism applied on the DM. First, we confirmed that the addition of these aberrations created no shift to the optical path length or the focal plane by tracking the axial pixel location of the cover glass surface on the spectral-domain (SD)-OCM images (Supplementary Fig. 3). Visually, apart from a few distortions, the SNR of the OCM images is sufficient to observe the cellular structures accurately. The similarity between these structures further shows that the intensity and lifetime were collected from the cells located in the same focal plane. As expected, the mean NAD(P)H fluorescence intensity of the frame decreases with an increase in the magnitude of OAs (Fig. 4c), which is also observed in the zoomed-in images in Fig. 4a at a single-cell scale. Besides the decrease in fluorescence intensity, the mean lifetime per frame is also shorter when the magnitude of OAs is larger (Fig. 4a,d), as expected due to the bias of low-SNR FLIM images towards a shorter lifetime value (Fig. 4b)^22–25^. This effect is corroborated by the number of pixels with a lifetime of 1500 ps or greater shown in Fig. 4e. The images with no aberrations report the cytosolic NAD(P)H lifetime more accurately, and close to previously reported values in cells^65^. The complete set of images is shown in Supplementary Fig. 4.

These results show that OAs affect the accuracy of measurements from FLIM. The images in Figs. 2 and 3 were acquired close to the surface of the tissue samples where the presence of OAs is often negligible and can be neglected. However, sample-induced OAs degrade the images obtained from deeper locations, especially acquired by FLIM. This is also observed in a homogenous sample like a beta barium borate (BBO) crystal, where the estimated fluorescence lifetime decreases deeper in the sample. Interestingly, there was no apparent trend in the changes to the fluorescence intensity and the scattering was minimal except for the refractive index mismatch between the homogenous BBO crystal and the immersion medium for the objective lens (water) that would distort the optical wavefront eventually causing significant aberrations in images from the deeper layers. (Supplementary Fig. 5). As seen in Fig. 4a, even when the OCM images do not show severe distortions or a severe decrease to the SNR making them indiscernible to the sample features, the accuracy of the measured lifetime is apparently reduced where the peak of the histogram of distribution of these lifetime values are different (Fig. 4b). We also observed this effect while imaging a cross-section of a rat kidney using FOCALS microscopy at two different depths – 5 µm and 45 µm below the surface (Fig. 4f–h). Figure 4f shows the multimodal images at 5 µm below the surface; the SHG signals were negligible. While the PS-OCM images appear to be nearly homogenously scattering with no discernable structures or polarization sensitivity, the renal corpuscular features are apparent in the FLIM image. The NAD(P)H fluorescence lifetime of the corpuscular structures is observed to be 1500–2500 ps. However, initial images of the kidney acquired at a flat profile of the DM highlight the bias towards shorter lifetime values as the SNR decreases^22–25^, particularly in the zoomed-in features that initially appear to have a NAD(P)H fluorescence lifetime of 1000 ps or shorter.

To correct these OAs we adapted a sensorless AO algorithm, called *AutoAO*, that we had previously demonstrated for OCT^51^. Unlike other commonly used sensorless AO algorithms that require a series of iterative images at different states of a DM, *AutoAO* requires just one initial volume for optimization that was acquired within 1.25 s. The algorithm iteratively optimizes for the CAO phase mask that minimizes an image metric on the GPU and translates this mask to the DM pattern that can correct these OAs (Supplementary Fig. 6). The initial publication described several versions of *AutoAO* for different applications and the volumetric aberration correction algorithm was utilized for FOCALS microscopy^51^. MPM images are highly sensitive to the location of the focal plane and the volumetric aberration correction algorithm ensures that there is no shift of the focal plane. In FOCALS microscopy, the *AutoAO* algorithm was executed for a volume of eight depths in OCM spread evenly across 30 µm around the focal plane, and took approximately 2 s to find the optimal focal plane. After aberration correction with *AutoAO*, the lifetime of the features in the rat kidney 45 µm below the surface were closer to the values on the surface (1500 ps or greater), indicating the presence of bound NAD(P)H (Fig. 4f–g). Although the improvement to the overall SNR was minimal, FOCALS microscopy with *AutoAO* using the pattern in Fig. 4h yielded a more accurate representation of the metabolic profile of the tissue, confirmed by the histogram (Fig. 4g) comparing the distribution of the estimated lifetimes.

The biological relevance of the improvement due to *AutoAO* is best exemplified by the FOCALS microscopy images of the murine gastrocnemius (calf) muscle shown in Fig. 4i–k. The mitochondrial reticulum in the muscle cells and the metabolic dynamics have been imaged and reported in several previous studies, based on which we expect the presence of bound NAD(P)H along the fibers^66,67^. The abundance of mitochondria along the myofibrils to form complex networks in myocytes has been previously reported^68^. In Supplementary Fig. 7, the 3D images of the muscle cell for depths between 0 and 17 µm below the surface, several bright spots are visible in the FLIM images with a NAD(P)H fluorescence lifetime of 1500 ps or longer along the myofibrils, indicating the presence of protein-bound NAD(P)H. However, as seen in Fig. 4i, at a depth of 50 µm below the surface, without *AutoAO* correction, these spots are sparse, as indicated by the dearth of orange dots overlaid on the OCM image (See Supplementary Fig. 2 for the OCM images without the overlaid dots). After *AutoAO* correction with the pattern shown in Fig. 4k, the total number of these spots is greater. The histogram showing the distribution of lifetimes shows that the OA-corrected images have longer mean lifetimes (Fig. 4j). Upon closer investigation of the locations indicated by the white arrows, these spots appear alongside the scattering structures in the muscle. Several of these spots, indicated by the magenta arrows, are embedded in the fibrous network imaged using SHG (Fig. 4i) and the bias towards a particular polarization state in the PS-OCM image. Although the OCM images appear similar before and after *AutoAO*, the improvement to the FLIM images revealed the metabolic profile of the muscle cells that was previously obscured by OAs even with small changes to the optical wavefront. These results establish the need for AO in quantitative fluorescence microscopy techniques in tissue imaging and the performance of the FOCALS microscopy setup for dynamic and 3D biological samples.

## Discussion

**Figure 5:**
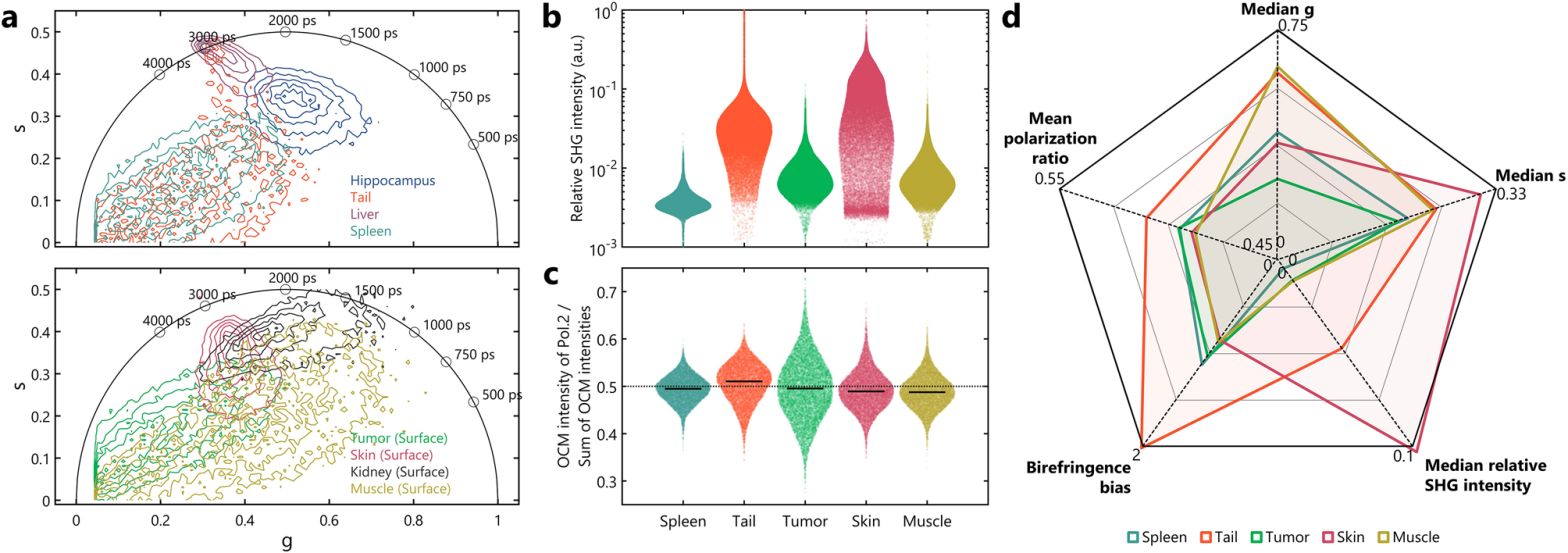
Tissue profiling with FOCALS microscopy. (**a**) Contour lines of the NAD(P)H autofluorescence lifetime data for the different tissues shown in Figs. 2, 3 and 4 on the phasor plot, where the threshold was set to be g > 0.05. (**b**–**c**) Beeswarm plots of the SHG intensity (normalized to 20 a.u. and in log scale) and the polarization ratio of pixels with SHG intensity greater than the 25th percentile of each frame. (**d**) Radar plot to show the multidimensional distribution of the quantities from FOCALS microscopy for the spleen, tail, tumor, skin, and muscle. The SHG intensities for the hippocampus, liver, and kidney were too weak for visualization or quantification; therefore, they were neglected in (**b**–**d**).

Having shown that FOCALS microscopy can image a diverse set of samples fast and accurately, we wanted to confirm the specificity of the combined modalities of FOCALS microscopy compared to the individual modalities. Figure 5a shows the distribution of the fluorescence lifetime on the phasor plots as contours for the different tissues imaged ex vivo and in vivo that were presented in Figs. 2, 3 and 4. The data had to be divided among two plots as there was too much overlap to distinguish the different samples visually. Samples with higher fluorescence intensity, such as the liver, skin, and kidney, have their contour centroids closer to the universal circle line. They also have a tighter distribution compared to the other weakly fluorescent samples like the spleen. The two distinct centroids for the tail are also apparent. Despite these subtle differences, the fluorescence lifetime alone is insufficient to profile these tissue samples. However, after extracting the other features from the SHG and PS-OCM images, further insight can be obtained (Fig. 5b–c). For instance, the SHG intensity distribution can report on the collagen content in the sample. The alignment of these fibrous structures can be discerned from quantifying the optical signatures from PS-OCM images (Fig. 5c). In Figs. 2, 3 and 4, the SHG images of the tail, muscle, and skin show aligned fibrous structures/bundles compared to the SHG images of the tumor where the collagen fibers are misaligned. This is reflected in the PS-OCM images where the polarization ratio, i.e. the ratio between the OCM intensity of Pol. 2 and the sum of OCM intensities, of the tumor and spleen are closer to 0.5 compared to the other samples where the aligned fibers induce birefringence. To isolate the effect of the fibers observed in SHG on the PS-OCM images, we devised a metric called birefringence bias, where the probability distribution of the polarization ratio of the pixels with high SHG intensity (above the 80th percentile of the frame) was fit to two different Gaussian functions on either side of the median. The ratio between the widths of these Gaussian functions can describe the alignment of these bright fibers in the SHG image. When all these metrics were observed cumulatively in the radar plot (Fig. 5d), there were several distinct shapes of the polygons for the different samples and the numerous points of intersection between them were apparent. These optical signatures presented in Fig. 5 were derived for the representative frames from a few fields of view for the samples presented in Figs. 2, 3 and 4; in this limited scope, these samples occupied unique spaces in the domain of multidimensional optical signals. These examples clearly demonstrate the capability of FOCALS microscopy to profile heterogeneous live samples and derive their unique multidimensional optical signatures. Nonetheless, we believe that this can be extended further to characterize live biological samples more accurately than the individual modalities for larger studies with focused applications.

Quantitative analysis of MPM images is increasingly used to describe the biochemical profiles of tissue samples. Most of the AO studies have been focused on improving the signal intensity at the focal plane with an intuitive understanding that better SNR would lead to more accurate quantitative images. We conclusively showed that even when the structures are not visibly distorted by OAs (in the tissue samples) or when the fluorescence intensity is sufficiently high and not changing with depth (in the BBO crystal), AO is necessary to quantitatively characterize these samples accurately. Currently, our AO performance is limited by the 52-element DM; this can be improved by switching to a DM with more elements, with a better linear response, and with the capability of patterning higher-order Zernike modes. While a part of the difference between the fluorescence lifetime at the surface and deeper in the kidneys (Fig. 4f) could be attributed to hypoxia deeper in the tissue due to lack of perfusion, the improvement to the fluorescence lifetimes due to *AutoAO* is nonetheless apparent, verified by the results from muscle imaging (Fig. 4i–k). As FLIM and MPM techniques become faster, the effect of OAs on the accuracy of the estimated lifetimes is expected to play a larger role than the inaccuracies due to sample dynamics within the exposure duration. In the context of metabolic imaging, ratios that connect the autofluorescence intensities of NAD(P)H and FAD have been used to describe the redox states of cancer cells and their microenvironments^9,69^. Typically, these ratios are compared against different samples or time points, assuming no local variations. However, if sample-induced OAs affect the photon counts from NAD(P)H and FAD differently, then the redox ratio could potentially be miscalculated. For instance, in multimodal systems with a 1050–1100 nm excitation source that can simultaneously induce two-photon excitation of FAD and three-photon excitation of NAD(P)H, the OAs could decrease the contribution of NAD(P)H to these ratios since three-photon excitation is comparatively weaker^9,57^. Therefore, similar to the improvement of the lifetime accuracy enabled by *AutoAO* in FOCALS microscopy, we plan to explore the role of AO for improving the accuracy of redox-ratio calculations in future studies.

Previous studies have reported the detrimental effect of long exposure times on estimated fluorescence lifetime values using traditional FLIM techniques^17,70^. Therefore, we needed a faster technique to observe the effects of OAs in dynamic samples. Thus the fast two-photon FLIM in FOCALS microscopy was adapted from a previous iteration of the setup^11^ with three key improvements. First, we realized that the need to acquire images with accurate autofluorescence lifetime values noninvasively exceeded the need for speed. The previous version of the setup used an 8-kHz resonant galvo mirror for fast scanning, with 10 pulses per pixel for each FLIM frame acquired at 25 frames per second (fps). Responses from 20 to 25 frames had to be averaged to determine NAD(P)H fluorescence lifetimes accurately. While maintaining the incident power below ANSI standards (Z136.3) (25–30 mW) and using the same laser source, we improved the excitation and detection efficiencies by using a pair of chirped mirrors for pulse compression, spatial filters to filter higher-order spatial modes of the laser source, and a PMT with a better quantum efficiency at 450 nm. Additionally, rather than averaging the response over 30 frames, the 8-kHz galvo mirror was replaced with one 30 × slower. For the results in Figs. 2, 3 and 4, the responses from 625 sequential excitation pulses were added together for a frame rate of 0.4 fps, instead of averaging the response over 63 frames. The effective frame rates for both configurations are similar (25 fps with each pixel in a frame containing the response from 10 pulses, averaged over 63 frames, is effectively 0.39 fps). The maximum permissible frame rate at the current pixel rate is approximately 1 Hz after accounting for galvo mirror “flyback”. The wait time between lines could be decreased to achieve this frame rate with slight improvements to the data pipeline efficiencies for longitudinal imaging. Second, adding the responses from sequential pulses also yielded better temporal stability. The synchronization was further improved by using an analog frequency divider to derive a 10-MHz reference clock from the 80-MHz laser and a phase-locked loop configuration to derive the line and frame clocks instead of pulse picking. (See Supplementary Table 1 for the description of all clocks and signals and Supplementary Table 2 for the image and acquisition parameters). Additionally, the transimpedance amplifier bandwidth was 1500 MHz; we doubled the input sampling rate from 1600 to 3200 MHz to prevent sub-Nyquist sampling by upgrading the digitizer. Third, moving to real-time phasor-based processing of FLIM images (described by Sorrells et al.^15^) reduced the effective data rate from 1.3 GBps to 210 kBps. (See Supplementary Table 3 for the list of all data sizes in FOCALS microscopy). Previous iterations of fast FLIM techniques capable of NAD(P)H autofluorescence imaging along with OCM were significantly slower (pixel rates of 78 Hz^71^, 30 kHz^52,53^, 128 kHz for FOCALS microscopy) and needed multiple light sources^52^. Currently, the speed of FOCALS microscopy is limited by the photon budget for NAD(P)H autofluorescence lifetime imaging; this can be improved by switching to lasers with a shorter pulse width or lower repetition rates. However, this speed was sufficient for in vivo imaging without any major artifacts. We had previously demonstrated in vivo FLIM imaging of murine skin using a typical TCSPC-based setup which took 51 s per frame, compared to the 2.5 s (and real-time processing) for FOCALS microscopy^54^. FOCALS microscopy also compares favorably against commercial FLIM imaging systems previously used for in vivo skin imaging^55,56^ with a 5–6 × improvement in imaging speed, respectively.

Based on the results in Figs. 2, 3, 4 and 5, FOCALS microscopy can differentiate between free and bound NAD(P)H in dynamic and 3D biological samples and correlate these to structural features. The stark difference in the lifetime profiles before and after AO is also apparent. These results suggest a need to investigate the depth-dependent accuracy of FLIM; it will be investigated in detail in future publications. Faster and higher bandwidth detectors or computational pulse picking techniques can be used to make the estimated values from FOCALS microscopy comparable to those derived from traditional FLIM techniques, which we will investigate in future studies.

## Conclusions

In this paper, the effect of OAs on FLIM was explored, which led to the realization and previously undiscovered need for AO and aberration correction for FLIM. We presented a fast and novel optical microscopy setup capable of generating FLIM, SHG, and PS-OCM images in real-time to specifically leverage the knowledge of OAs in OCT and OCM, and extended these to MPM and FLIM studies with high-speed performance for dynamic imaging applications. In addition, we presented FOCALS microscopy images of a diverse variety of samples ranging from cell-culture models, to healthy neural, muscular, renal, hepatic, and gastric microenvironments, to the 3D ex vivo tumor microenvironment, and to in vivo skin to highlight the features of FOCALS microscopy. Finally, we emphasized the role of *AutoAO* to enable accurate estimation of metabolic profiles from deeper regions in the tissue samples. In ongoing and future studies, we plan to use this setup for investigating the fast neurometabolic changes during action potentials and neural circuit communications, for observing cancer dynamics, and for bacterial metabolic imaging in real-time, both ex vivo and in vivo. The multimodality, speed, and versatility of FOCALS microscopy enable it to be an effective tool for comprehensive investigations of life in action.

## Methods

### FOCALS microscopy setup

The FOCALS microscopy setup (Fig. 1) was sourced by a titanium-sapphire laser (MaiTai 780 HP, Spectra-Physics, Milpitas CA) which was successively reflected 15 times by a pair of chirped mirrors (UMC10-15FS, Thorlabs Inc., Newton NJ) to generate a pulse-width of 220 fs at 760 nm just before the objective lens. A small part of this beam was directed into a photodiode (PDB450A, Thorlabs Inc. operated at 150 MHz) to detect the laser clock. We used 750 nm for imaging the MDA-MB-231 cell dynamics. A part of the remaining beam (600 mW) was split off to generate the supercontinuum for OCM. The other part of the beam was sent through an HWP and a Glan–Thompson polarizer (GTH5-A, Thorlabs Inc.) to attenuate the beam. The attenuated beam was focused and passed through a 100-µm pinhole to filter out the higher-order spatial modes and was then collimated. The transverse resolution measured using fluorescence beads (for FLIM) was 0.4 µm. The excitation beam was expanded to fill the aperture of the DM (Mirao 52e, Imagine Optics, Orsay, France). The beam size was reduced and passed through a pair of galvo mirrors (6230H, Cambridge Technology, Bedford MA) in a telecentric configuration. The objective lens (XLPLN25XWMP2, Olympus Corporation, Waltham MA) was placed such that the back focal plane of the objective was conjugated with each galvo mirror and the DM. Similarly, the sample plane was conjugated with both PMTs. The FLIM PMT (H10721-210, Hamamatsu Photonics, Bridgewater NJ) was connected to a custom 5-V power supply (LC7805 powered by a 9 V AC-DC adapter) with resistance programming to generate a control voltage of 1.05 V. The PMT was connected to a 1500-MHz-bandwidth TIA unit (C5594, Hamamatsu Photonics). The output of the amplifier was attenuated through a 16-dB attenuator and sent to the input of a high-speed 12-bit digitizer (ATS9373, AlazarTech Technologies Inc., Pointe-Claire, Canada) operated at a sampling rate of 3.2 gigasamples per second. The emitted nonlinear signal was collected in reflection mode through a dichroic to reflect shorter wavelengths (FF665-Di01-25 × 36, Semrock, IDEX Health & Science LLC, Rochester, NY), passed through two 633-nm short-pass filters to prevent any bias from back-reflected excitation light in lifetime estimation (BSP01-633R-25, Semrock), and further split into the FLIM and SHG signals through an optional dichroic. The results in Figs. 2, 3, 4 and 5 were acquired with a 450 ± 70 nm filter (FF01-450/70, Semrock) placed in front of the FLIM PMT and a dichroic mirror with a cutoff wavelength at 390 nm (FF01-380/LP, Semrock) to send the SHG signals to the other PMT. No filter was used in front of the SHG PMT (H7421-40, Hamamatsu Photonics) to maximize the signal collection efficiency. However, the drawbacks of this can be noted in Fig. 2f, where the bright features appearing in the FLIM images are also visible in the SHG channel due to signal leakage. The output of the SHG PMT was connected to one of the input channels of a data acquisition (DAQ) card (PCIe 6323, National Instruments, Austin TX), externally available through a pair of terminal blocks (BNC 2110, National Instruments). The zero-frequency component of the laser clock was filtered using a 10 µF capacitor and sent to the sinusoidal input pin of a 2-phase negative emitter-coupled logic (NECL)/ transistor-transistor logic (TTL) frequency divider (PRL-260BNT, Pulse Research Lab, Torrance, CA). The TTL output of the divider was connected to a 1:4 TTL fanout buffer (PRL-414B, Pulse Research Lab) to generate two TTL 10 MHz signals, sent as external reference clocks to the digitizer and the DAQ card.

The source for PS-OCM was generated using the 600 mW beam split off the laser output to pump a photonic crystal fiber (PCF) (LMA-PM-5, Thorlabs Inc.). The PCF output was collimated with a parabolic mirror and refocused into a single-mode patch cable (780 HP, Thorlabs Inc.) to obtain a Gaussian beam profile. The output of the single-mode fiber was collimated, passed through a quarter-wave plate (QWP) to obtain a circularly polarized beam, and split into sample and reference beams using a beamsplitter cube. The sample arm beam was combined with the FLIM excitation beam before they are expanded to fill the aperture of the DM. At 760 nm, the bandwidth at the output of the PCF was 80 nm, corresponding to an axial resolution of approximately 3.2 µm in air; the transverse resolution measured using TiO_2_ particles embedded in silicone was 0.7 µm (for OCM). The reference beam for OCM contained a polarization-dependent delay loop where the optical path lengths were different for each polarization state. Therefore, the polarization-multiplexed signal was detected using a single spectrometer (Cobra 800, Wasatch Photonics, Morrisville NC)-line scan camera (Sprint spL4096-140-km, Basler Inc., Exton PA) connected to a frame grabber (PCIe 1433, National Instruments Corp.). An RTSI cable was used to synchronize the clocks between the DAQ card and the frame grabber). A 3D sample stage (VP-25XA-XYZR, Newport Corp., Milpitas CA) was used to find the optimal location for imaging in the sample. The stage was turned off during *AutoAO* to ensure phase stability. The complete set of clocks and signals and their descriptions are provided in Supplementary Table 1. The incident power at the sample plane was maintained at 30 mW for all biological samples, 1 mW for the urea crystals, and 0.3 mW for the BBO crystals. The SHG of the BBO crystals was acquired with a bandpass filter centered at 380 nm (FF01-379-34, Semrock, IDEX Health and Science) at 760-nm excitation without the dichroic mirror that separates the FLIM and SHG signals to ddirect all the emitted nonlinear signals from the BBO crystal into the analog PMT through the filter.

### Data acquisition and processing

Data were acquired using a custom LabVIEW software (see Supplementary Movie 2 for a screen capture of the acquisition and real-time processing software imaging NE-4C cells and Supplementary Fig. 8 for a screenshot of the MDA-MB-231 cells images with FLIM and PS-OCM) implemented as a state machine diagram with separate data queues for displaying and saving each modality. The data transfers for OCM and FLIM were staggered on the rising and falling edges of the clock, respectively, which caused the OCM image to be circularly shifted by one line in each frame. The code for streaming data from the digitizer was written in C and compiled as a dynamic linked library. Similarly, the code for real-time processing was written in C using CUDA libraries, with separate dedicated streams for OCM and FLIM processing, and compiled as a dynamic linked library. The complete description of the FLIM processing code and characterization of the FLIM in FOCALS microscopy using fluorescent standards were presented in Sorrells et al^15^. Briefly, phasor analysis of FLIM involves finding the Fourier series coefficients for the sine and cosine components (usually denoted as s and g, respectively) at the modulation frequency, i.e., 80 MHz. The mean lifetime was therefore proportional to the ratio between s and g assuming that the response to each excitation is modelled as a single exponential decay. It should be noted that the lower limit of the amplifier bandwidth is 50 kHz, which may cause shifts to the DC value over 1600 pulses if there are a significantly large number of photons. However, considering the signal levels from biological samples used in the paper and that each pixel (corresponding to 625 laser pulses) is processed independently with DC value subtraction as described by Sorrells et al﻿^15^, the resultant FLIM measurements are not affected.

Each frame of FLIM contained 256 × 256 pixels spanning 128 × 128 µm^2^. Each pixel represented the response from 625 excitation pulses corresponding to a pixel rate of 128 kHz. This was the same as the line-scan rate for OCM acquisition, where 896 pixels were acquired along the spectral axis for each A-scan. The data was acquired in batches or ‘chunks’, i.e. a small wait time of 4 A-scans was inserted between every 32 A-scans acquired to prevent gridlocks in data bottlenecks (see Chunk clock in Supplementary Table 1). All raw data were processed on the GPU using an executable version of the same processing code to generate the images for display. All FLIM images were displayed after 3 × 3 median filtering, where the false-colored lifetime images were overlaid on a transparency map corresponding to the fluorescence intensity.

The OCM data was converted into space-domain after dispersion compensation, k-space resampling, and inverse Fourier transform, similar to the procedure described in Iyer et al^72^. The PS-OCM ratios were overlaid on a transparency map corresponding to the average OCM intensity of the two polarization states. The images indicated as ‘Pol.’ in Figs. 2, 3, 4 and 5 are the polarization ratio expressed as the ratio between the intensity of polarization state 2 and the sum of the two OCM intensity states. The two intensities were scaled such that a surface of a coverslip had a ratio of 0.5. This calibration was done before the beginning of each OCM imaging session. See Supplementary Fig. 9a for the images of the polarization ratio of urea crystals acquired with a half-wave plate placed before the fast galvo mirror. As expected, highly-birefringent urea crystals are highly heterogeneous the median ratio follows a sinusoidal pattern when the half-wave plate is rotated at different angles. Supplementary Fig. 9b shows images of the glass coverslip with different magnitudes of aberrations imparted at the DM to show the distribution of the polarization-ratio (which is ideally 0.5) and the stability of calibration.

The birefringence bias was calculated from the probability distribution of the polarization ratio of the pixels with high SHG intensity (above the 80th percentile of the frame), which was fit to two different Gaussian functions on either side of the median. The ratio between the widths of these Gaussian functions was used to describe the alignment of these bright fibers in the SHG image. The perceptually accurate colormaps were chosen specifically to avoid visual bias due to uneven brightness^73^. All quantitative analysis on data and the generation of the figures for display in this manuscript was done in MATLAB 2021a (Mathworks Inc., Natick MA). Any pixels with g or s components below 0 were set to have a transparency of 0. Any pixel with a fluorescence intensity less than the 5th percentile was discarded from any quantitative analysis. All SNRs were calculated as the ratio between the 99th and 5th percentile of intensity values in the frame.

The *AutoAO* algorithm was previously described in Iyer et al^51^. Previously we had used a sharpness metric defined by Eq. (1).

1 $$\mathop{\arg \max}\limits_{{W_{{_{m}}}^{n}}} {\text{sign}}\left({\gamma - 2} \right)\;\sum\limits_{x,y,z} {\left| {E_{CAO} \left({x,y,z} \right)} \right|^{\gamma}}.$$

However, we devised a new Fourier metric to minimize the bias against sample structures consisting of four parts.

2 $$\mathop{\arg \max }\limits_{{W_{{_{m} }}^{n} }} \Psi_{0} + c_{1} \Psi_{1} + c_{2} \Psi_{2} + c_{3} \Psi_{3}$$

3 $$\begin{aligned} \Psi _{0} & = - 20\log _{{10}} \sum\limits_{{k_{x} ,k_{y} ,z}}{\frac{{\left| {M_{{k_{r}>f_{{high}}}} \left({\widetilde{E}_{{CAO}} \left({k_{x} ,k_{y} ,z} \right)} \right)} \right|}}{{\left| {M_{{k_{r} < f_{{low}} }} \left({\widetilde{E}_{{CAO}} \left( {k_{x} ,k_{y} ,z} \right)} \right)} \right|}}} \; \\ \Psi _{1} & = - 20\log _{{10}} \sum\limits_{{k_{x} ,k_{y} ,z}} {\frac{{\left| {M_{{k_{r} > f_{{high\;x}} \& k_{\theta } \in \left\{[\frac {-{\pi}}4, \frac {{\pi}}4  ] ,  [\frac {3{\pi}}4, \frac {5{\pi}}4 ]\right\}}} \left({\widetilde{E}_{{CAO}} \left( {k_{x} ,k_{y} ,z} \right)} \right)} \right|}}{{\left| {M_{{k_{r} > f_{{high\;x}} \& k_{\theta} \in \left\{[\frac {{\pi}}4, \frac {3{\pi}}4  ] ,  [\frac {5{\pi}}4, \frac {7{\pi}}4 ]\right\}}} \left( {\widetilde{E}_{{CAO}} \left( {k_{x} ,k_{y} ,z} \right)} \right)} \right|}}} \\ \Psi _{2} & = - 20\log _{{10}} \sum\limits_{{k_{x} ,k_{y} ,z}} {\frac{{\left| {M_{{k_{r} > f_{{high\;x}} \& k_{\theta } \in \left\{[{0}, \frac {{\pi}}2  ] ,  [ {{\pi}}, \frac {3{\pi}}2 ]\right\}}} \left( {\widetilde{E}_{{CAO}} \left({k_{x} ,k_{y} ,z} \right)} \right)} \right|}}{{\left| {M_{{k_{r} > f_{{high\;x}} \& k_{\theta } \in \left\{[\frac {{\pi}}2, {{\pi}}  ] ,  [\frac {3{\pi}}2, {2{\pi}} ]\right\}}} \left( {\widetilde{E}_{{CAO}} \left( {k_{x} ,k_{y} ,z} \right)} \right)} \right|}}} \\ \Psi _{3} & = \sum\limits_{{k_{x} ,k_{y} ,z}} {\left| {M_{{k_{r} > f_{{high}} }} \left( {\widetilde{E}_{{CAO}} \left( {k_{x} ,k_{y} ,z} \right)} \right)} \right|} \\ \end{aligned}$$

4 $$\widetilde{E}_{CAO} \left( {k_{x} ,k_{y} ,z} \right) = \user2{\mathfrak{F}}_{{x \to k_{x} ,y \to k_{y} }} \left\{ {\left| {E_{CAO} \left( {x,y,z} \right)} \right|} \right\}$$

5 $$E_{CAO} \left( {x,y,z} \right) = \user2{\mathfrak{F}}_{{k_{x} \to x,k_{y} \to y}}^{{^{ - 1} }} \left\{ \begin{gathered} \user2{\mathfrak{F}}_{{x \to k_{x} ,y \to k_{y} }} \left\{ {E_{OCM} (x,y,z)} \right\} \hfill \\ e^{{ - j\left( {\sum\limits_{{m,n|\left\{ {m,n} \right\} \ne \{ 2,0\} }} {W_{n}^{m} Z_{n}^{m} \left( {k_{x} ,k_{y} } \right)} + W_{Defocus} \left( {z - z_{foc} } \right)Z_{2}^{0} \left( {k_{x} ,k_{y} } \right)} \right)}} \hfill \\ \end{gathered} \right\}$$

6 $$\begin{aligned} & \widehat{M}_{condition} = \left\{ {\begin{array}{*{20}l} 1 & {{\text{if}}\;{\text{condition}}} \\ 0 &\quad {{\text{otherwise}}} \\ \end{array} } \right.  \\ & M_{condition} = \frac{{\widehat{M}_{condition} }}{{\sum {\widehat{M}_{condition} } }}  \\ \end{aligned}$$

Here, *E*_*OCM*_ is the complex-valued OCM images in 3D. *E*_*CAO*_ is the complex-valued OCM images in 3D after computational correction of the OAs using the phase mask, defined as

7 $$\phi\left( {k_{x} ,k_{y} } \right)=\sum\nolimits_{{m,n|\left\{ {m,n} \right\} \ne \{ 2,0\} }} {W_{n}^{m} Z_{n}^{m} \left( {k_{x} ,k_{y} } \right)} + W_{Defocus} \left( {z - z_{foc} } \right)Z_{2}^{0} \left( {k_{x} ,k_{y} } \right),$$

in the 2D Fourier plane defined as a sum of weighted Zernike polynomials, where the *W* terms are the coefficients and the *Z* terms are the polynomials. The defocus term was excluded from the optimization settings to ensure that there is no shift to the focal plane to avoid spatial dislocation of the MPM images after maximization and the average displacement of the pattern was maintained at 0 by using the piston. The term Ψ_1_ balances the ratio between high and low frequency components. Ideally, the ratio would be close to 1, containing equal amounts of high and low frequency components. However, to avoid optimization bias along certain directions (through unwanted addition of astigmatism or coma), two regularization terms, Ψ_2_ and Ψ_3_, were added to balance the frequency components along the 45°-135° axes and the 0°-90° axes, respectively. Finally, to maximize the image sharpness, a third regularization term, Ψ_4_, was introduced. The binary masks, *M*_*condition*_*,* in the Fourier plane are normalized such that the sum of each mask equals 1. The DM patterns are similarly described as the sum of Zernike polynomials, $\sum {A_{m}^{n} } Z_{m}^{n} \left( {x^{\prime},y^{\prime}} \right)$. The translation between the CAO phase masks and the DM patterns following the same procedure described in Iyer et al^51^. and the resulting matrix is shown in Supplementary Fig. 4. In FOCALS microscopy, we calculated both the CAO phase mask and DM patterns for defocus, primary spherical aberration, oblique and vertical astigmatism, horizontal and vertical coma, and oblique and vertical trefoil.

For the results in Fig. 4a, the astigmatic patterns were generated by varying $Z_{2}^{ - 2}$ and $Z_{2}^{2}$. The magnitude of aberrations was defined as $\sqrt {\left( {Z_{2}^{ - 2} } \right)^{2} + \left( {Z_{2}^{2} } \right)^{2} }$ and the angle of astigmatism as ${\text{atan2}}\left( {Z_{2}^{ - 2} ,Z_{2}^{2} } \right)$. While other studies have used alternative expressions for astigmatism that make the patterns symmetric about the 0°–180°-axis, we chose this representation to highlight the anisotropy in the multiphoton characteristics. The angle of astigmatism was fixed at 45°. All DM patterns are expressed in µm, proportional to voltages applied to each element. The actual pattern may vary slightly based on the response of the continuous surface of the DM.

### Cell culture

Secondary cultures of NE-4C mouse neuroectodermal cells (CRL-2925, American Type Culture Collection, Manassas, VA, USA) were plated on a 35-mm glass-bottom Petri dish with a cell adherent coating and grown in Eagle’s modified essential medium with a total of 4 µm L-glutamine (10009CV, Corning, Corning, NY, USA), supplemented with 10% v/v fetal bovine serum (16140071, Thermo Fisher Scientific, Waltham, MA, USA) and 1% v/v Penicillin–Streptomycin (10378016, Thermo Fisher Scientific) for 30 h in an incubator at 37 °C with 95% air and 5% CO_2_. The cells were imaged at room temperature within 10 min of being taken out of the incubator.

MDA-MB-231 cells (ATCC HTB-26, American Type Culture Collection) were grown in a 75-cm^2^ flask with a cell adherent (BioLite™, Thermo Fisher Scientific, Waltham, MA) coating in a phenol red-free media comprised of Dulbecco’s modified Eagle medium with high glucose and sodium pyruvate (12800017, Thermo Fisher Scientific) supplemented with 10% v/v fetal bovine serum (16140071, Thermo Fisher Scientific, Waltham, MA, USA) and 1% v/v Penicillin–Streptomycin (10378016, Thermo Fisher Scientific). The cells were trypsinized by topical application and centrifuged at 125×*g* for 5 min. After removing the supernatant, the pellet was dissolved in 2 mL of pre-warmed culture media. After finding the focal plane, 100 µL of the solution containing the cells was added to an empty 35-mm glass-bottom culture dish with a cell adherent poly-d-lysine coating (P35GC-0-14-C, MakTek Corporation, Ashland MA) placed at the sample plane during acquisition. The cells were imaged at room temperature within 10 min of resuspending the pellets at room temperature.

### Tumor induction, extraction, and imaging

All animal procedures were conducted in accordance with protocols approved by the Illinois Institutional Animal Care and Use Committee at the University of Illinois at Urbana-Champaign. All experiments in this study were carried out in compliance with the ARRIVE guidelines. Approximately 9 × 10^6^ MAT B III rat mammary adenocarcinoma cells (CRL-1666, American Type Culture Collection) were grown in the same media comprised of McCoy’s 5A (Modified) medium (16600082, Thermo Fisher Scientific) supplemented with 10% v/v fetal bovine serum (16140071, Thermo Fisher Scientific, Waltham, MA, USA) and 1% v/v Penicillin–Streptomycin (10378016, Thermo Fisher Scientific), and were injected subcutaneously into a healthy rat. After 7 days, when the tumor was approximately 5 × 5 × 2 mm^3^, the rat was euthanized by isoflurane overdose. Approximately 15 min postmortem, the tumor was surgically resected and placed in an imaging dish containing cold saline.

### Ex vivo tissue extraction and imaging

Healthy mice and rats were euthanized with CO_2_ asphyxiation. The tissues were then surgically resected and imaged in an imaging dish containing cold saline. The hippocampal slice was approximately 500 µm thick. One of the liver lobes was bisected with the flat surface placed in contact with the coverslip. The skin from a section of the tail was peeled to expose the collagen fibers and the vasculature for imaging.

### In vivo skin imaging

Most of the hair was removed in a 1 cm^2^ region in the dorsal caudal region of a 1-year old mouse (C57BL/6J) along the midline under 3% isoflurane anesthesia. The mouse was placed on the sample stage on a clean glass coverslip. A 1.5% isoflurane-O_2_ gas mixture delivered via nosecone was used for anesthesia and was maintained throughout imaging; a heating pad was placed on the mouse to maintain body temperature. Imaging lasted up to 1.5 h from the onset of anesthesia. Respiration rate was monitored throughout the imaging session by observing the imaging artefacts from breathing. The mouse was euthanized at the end of the imaging session via decapitation under deep sedation.

All methods were carried out in accordance with relevant guidelines and regulations.

## Supplementary Information

Supplementary Information 1.

Supplementary Video 1.

Supplementary Video 2.

## Data Availability

The data that support the findings of this study are available from the corresponding author upon reasonable request and through collaborative investigations.

## Code availability

The codes that were used to generate the quantitative results in this manuscript (e.g. analysis of cell trajectories) are available from the corresponding author upon reasonable request and through collaborative investigations.
