# Supplementary material for: Label-free metabolic and structural profiling of dynamic biological samples using multimodal optical microscopy with sensorless adaptive optics
Source: Sci Rep. 2022 Mar 2;12:3438. doi: 10.1038/s41598-022-06926-w (PMC8891278; doi:10.1038/s41598-022-06926-w)
Supplement: Supplementary file 1 — Supplementary Information 1. [file 41598_2022_6926_MOESM1_ESM.pdf]

Supplementary information for "Label-free metabolic and structural profiling of dynamic biological samples using multimodal optical microscopy with sensorless adaptive optics"

Rishyashring R. Iyer[1,2], Janet E. Sorrells[1,3], Lingxiao Yang[1,2], Eric J. Chaney[1], Darold R. Spillman Jr.[1], Brian E. Tibble[1,4], Carlos A. Renteria[1,3], Haohua Tu[1,2], Mantas Žurauskas[1], Marina Marjanovic[1,3], Stephen A. Boppart[1,2,3,5,6,*]

[1]Beckman Institute for Advanced Science and Technology, University of Illinois at Urbana-Champaign, Urbana, USA

[2]Department of Electrical and Computer Engineering, University of Illinois at Urbana-Champaign, Urbana, USA

[3]Department of Bioengineering, University of Illinois at Urbana-Champaign, Urbana, USA

[4]The School of Molecular and Cellular Biology, University of Illinois at Urbana-Champaign, Urbana, USA

[5]Carle Illinois College of Medicine, University of Illinois at Urbana-Champaign, Urbana, USA

[6]Cancer Center at Illinois, University of Illinois at Urbana-Champaign, Urbana, USA

*Corresponding author: boppart@illinois.edu

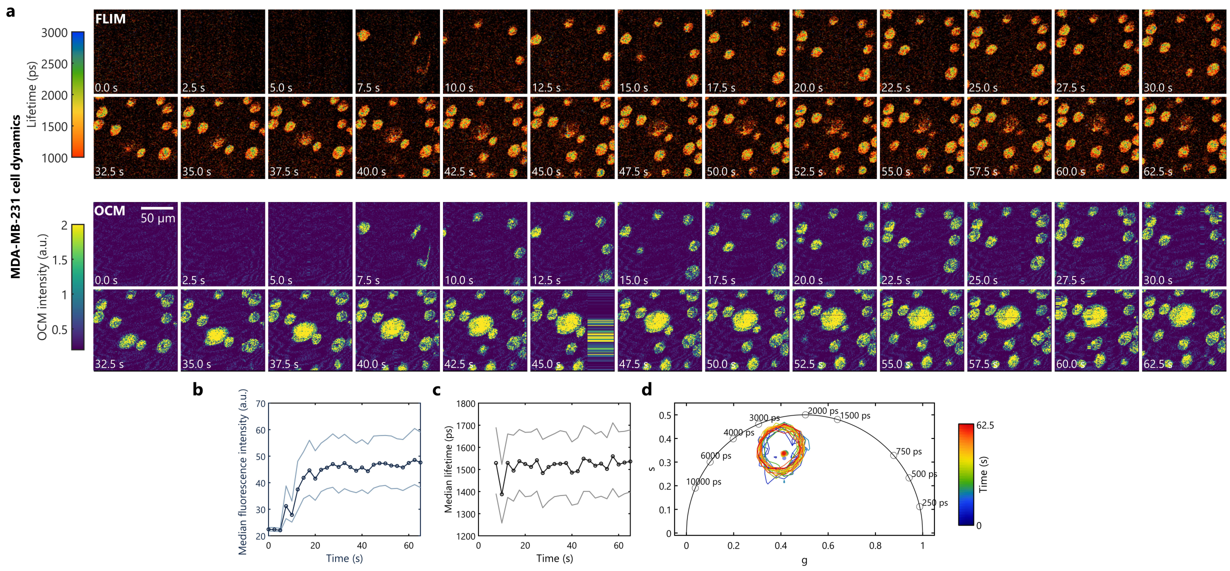

Supplementary Figure 1. FOCALS microscopy can image the metabolic dynamics of cells attaching to a glass surface quantitatively on both FLIM and OCM simultaneously.

a. Filmstrip of FLIM and OCM images of MDA-MB-231 cells dropped onto a glass surface acquired at a rate of 0.4 frames per second to track the arrival and motion of individual cells between successive frames on both imaging modalities (FLIM and OCM). The video is shown in Supplementary Movie 1 along with accompanying plots to track the changes to the mean OCM and MPM intensities, and the mean NAD(P)H fluorescence lifetime. **b.** Median fluorescence intensity of the frame in dark blue with the 40% and 60% quantile intensities in light blue. **c.** Median fluorescence lifetime of the frame in dark blue with the 40% and 60% quantile intensities in light blue. **d.** Phasor analysis of each frame shown as contour plots, where the shift in the trend to the overall lifetime can be observed by tracking the gradual shift from blue (t = 0 s) to red (t = 62.5 s).

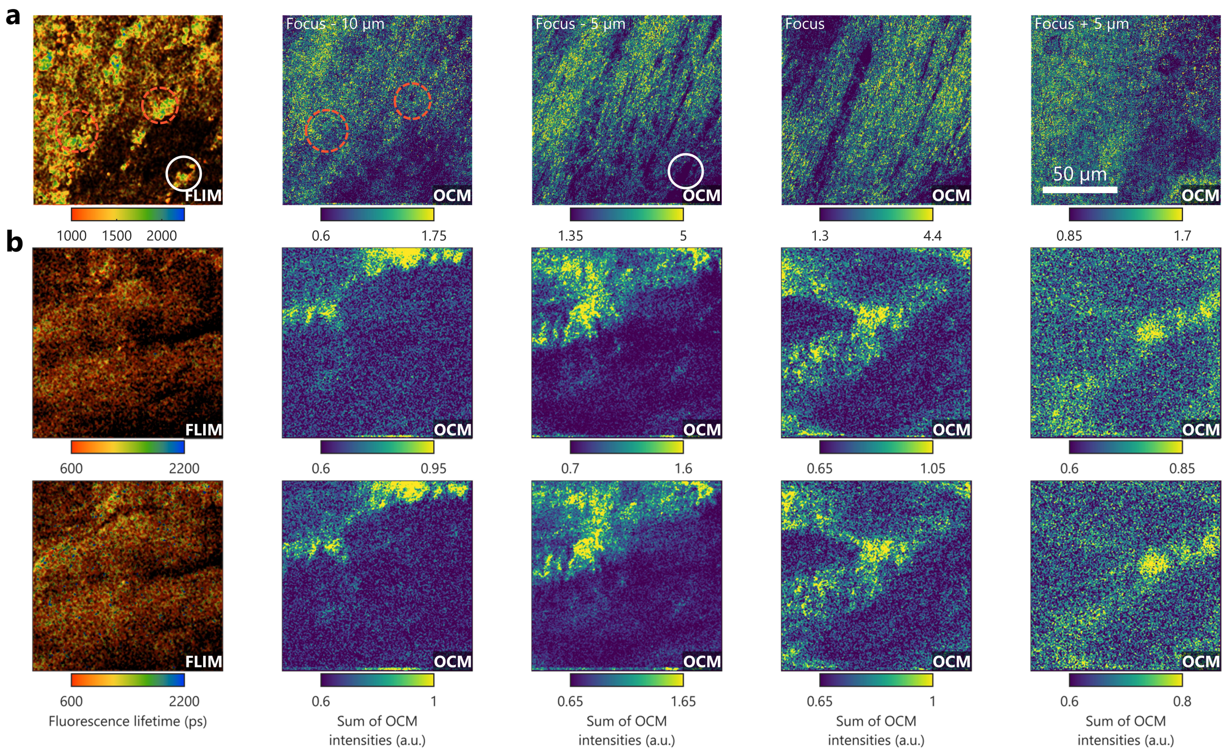

Supplementary Figure 2. The structures in FLIM can be tracked in the *en face* OCM images across a 20 µm region around the focal plane of the a. hippocampus and b. muscle (corresponding to Fig. 2d and 4i in the main manuscript)**.** The structures highlighted in orange and white correspond to the cell-like structures observed in FLIM. The structures highlighted in orange have strong fluorescence signals and have strong OCM scattering, the white structures appear as gaps between the fibers the OCM image despite having strong autofluorescence.

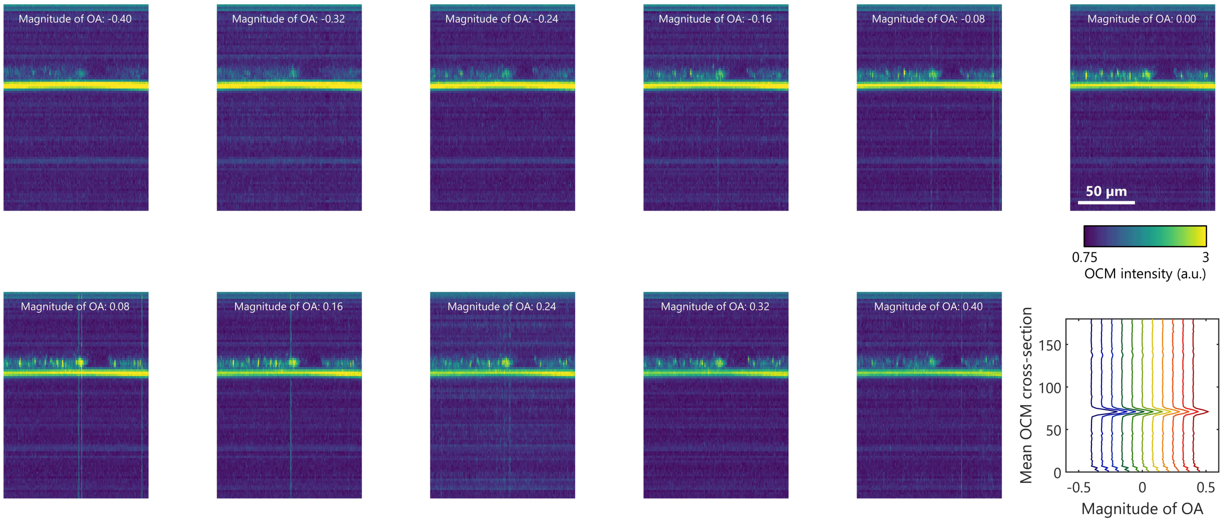

Supplementary Figure 3. Tracking the axial location of the coverslip at different aberration states shows that the patterns applied to the DM do not cause any changes to the overall optical path length. The OCM cross section shown as the maximum intensity projected over the fast axis showing the NE-4C cells on a coverslip with different magnitudes of astigmatism imparted at the deformable mirror. The adjacent graph shows the mean intensity of each of these cross-sections along the horizontal dimension to show that the axial location of the coverslip does not change with changing patterns on the deformable mirror.

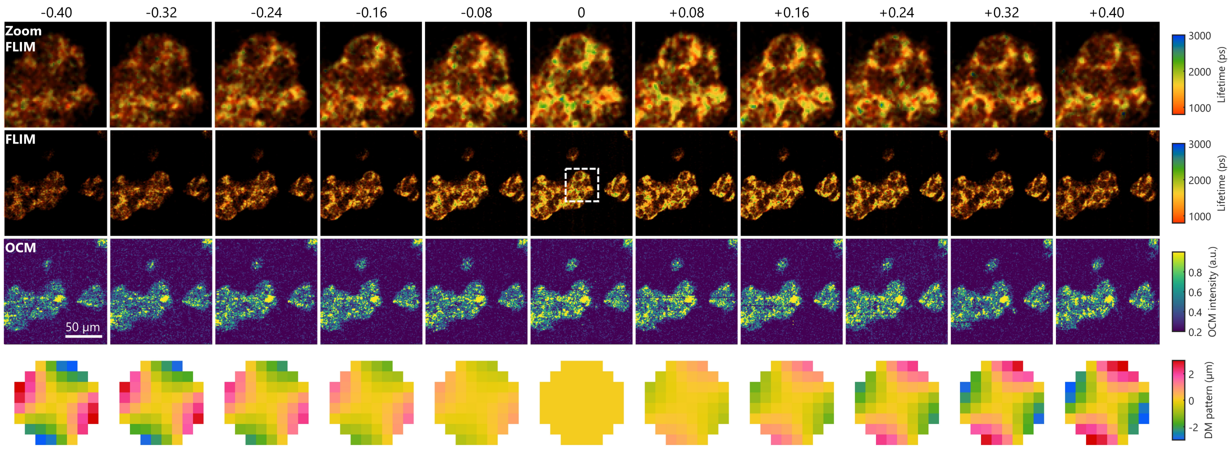

Supplementary Figure 4. Addition of optical aberrations changes to the measured fluorescence lifetime values, as shown on the NE-4C cells. FLIM, OCM, and corresponding DM patterns by varying the magnitude of the astigmatism coefficients between -0.08 to 0.08, while keeping the direction constant at 45°. The zoomed-in images correspond to the region highlighted by the white box.

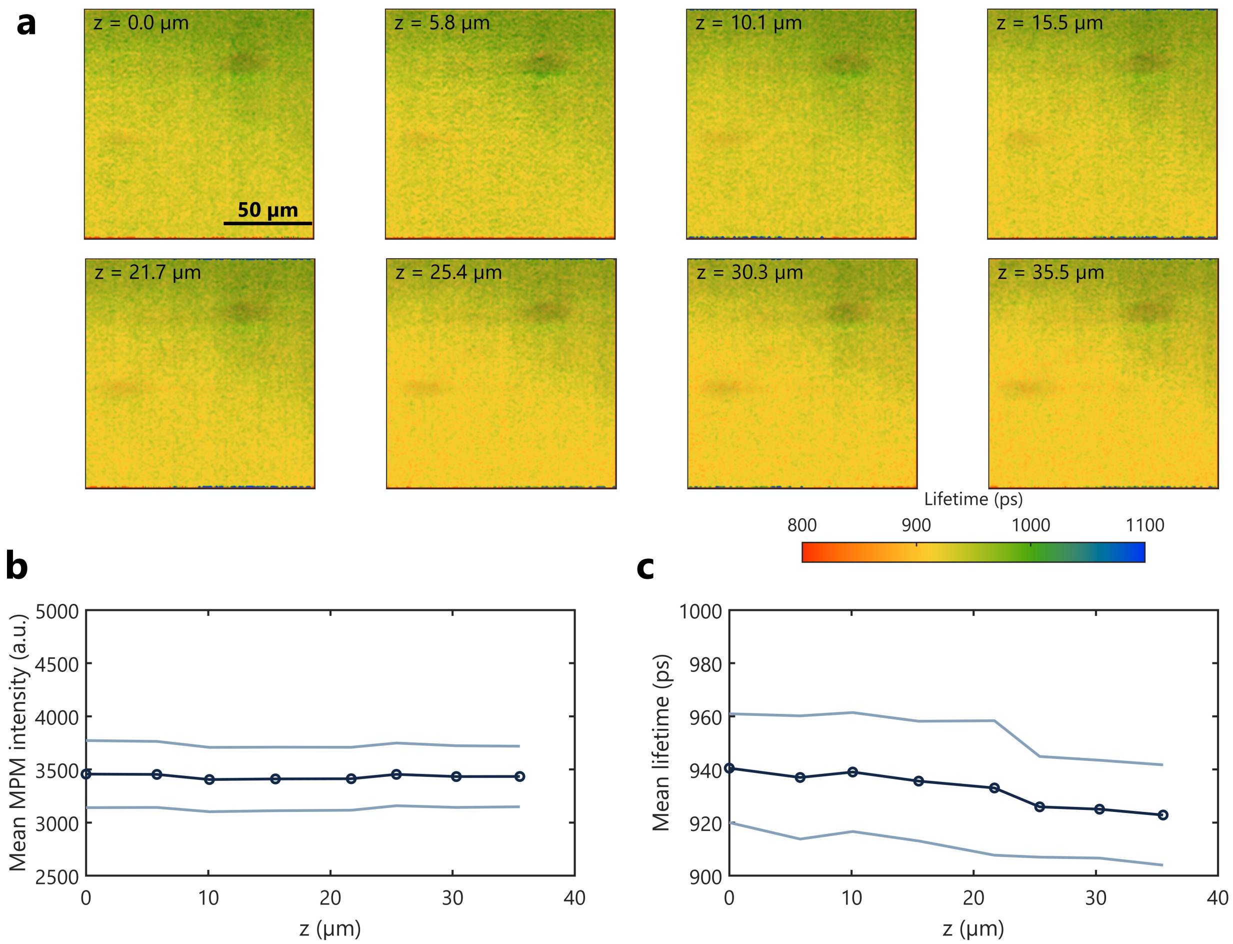

Supplementary Figure 5. In a homogeneous sample, deeper imaging creates minimal changes to the estimated lifetimes, compared to heterogeneous samples. a. A beta barium borate crystal imaged at different depths. **b.** Mean MPM intensity of the frame at different depths. **c.** Mean lifetime per frame at different depths. The light blue lines indicate ± standard deviation with respect to the mean.

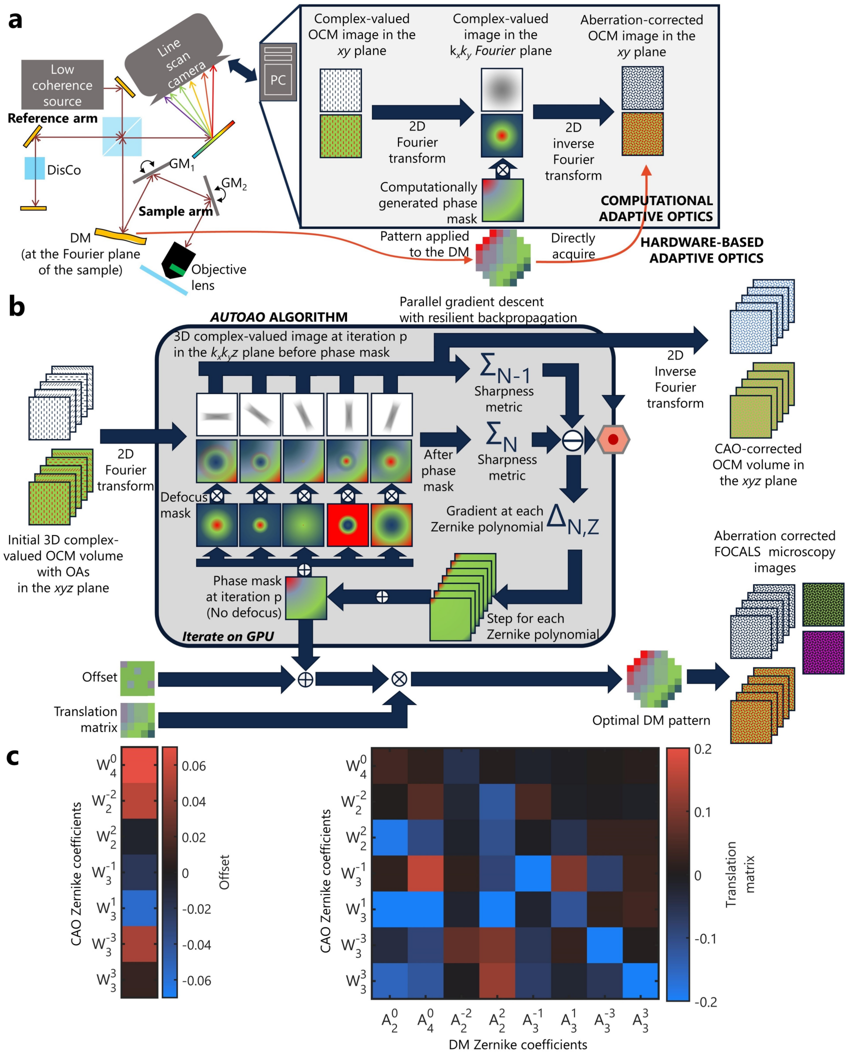

Supplementary Figure 6. Sensorless adaptive optics in FOCALS microscopy was achieved using the *AutoAO* algorithm. a. Brief schematic of computational adaptive optics (CAO) and hardware-based adaptive optics (HAO) using a DM, illustrated for a typical spectral-domain optical coherence microscopy (OCM) setup. **b.** Illustration and processing flowchart of the *AutoAO* algorithm. **c.** The offset and translation matrix to convert the Zernike coefficients (W_m^n) of the CAO phase mask to the Zernike coefficients (A_m^n) of the DM pattern.

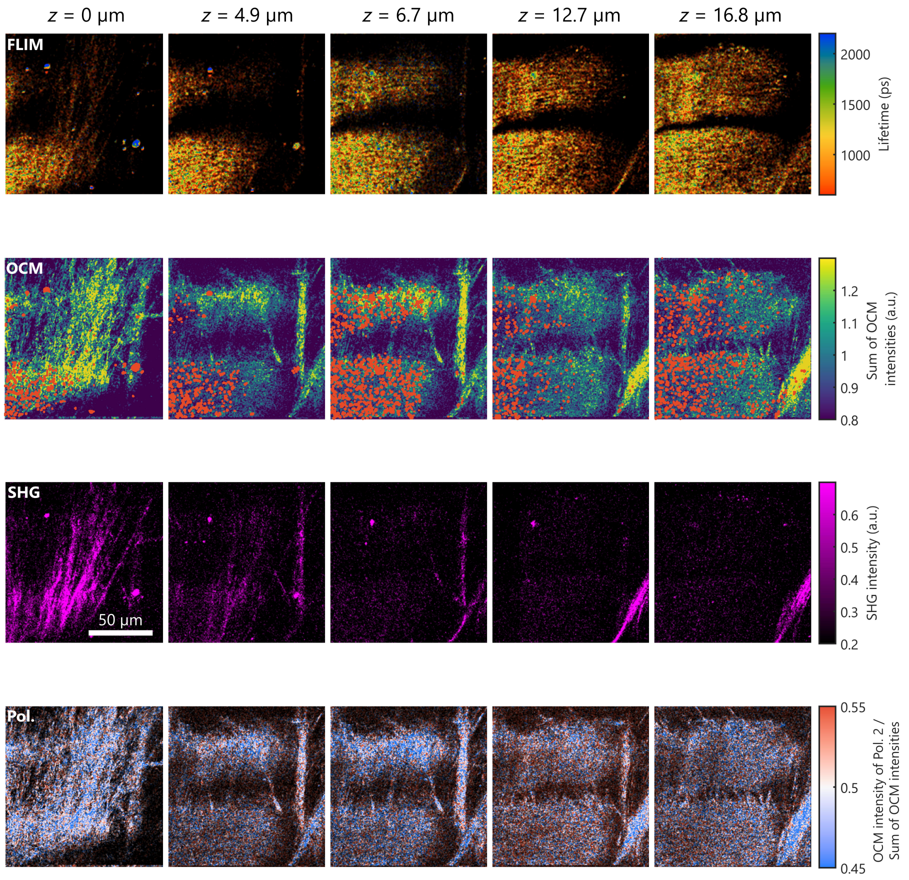

Supplementary Figure 7. 3D FOCALS microscopy images of a mouse calf muscle near the surface shows the presence of several pixels with high fluorescence lifetimes. FLIM, OCM, SHG, and PS-OCM microscopy images at 5 different depths over 17 μm. The orange dots in the OCM images indicate pixels whose lifetime is 1500 ps or longer. The OCM images show the scattering due to both the cells and the collagen fibers in the environment. They can be distinguished by tracking individual collagen fibers the SHG images and the cellular structures on the FLIM images. The characteristic striations of muscle cells are also apparent in the FLIM images.

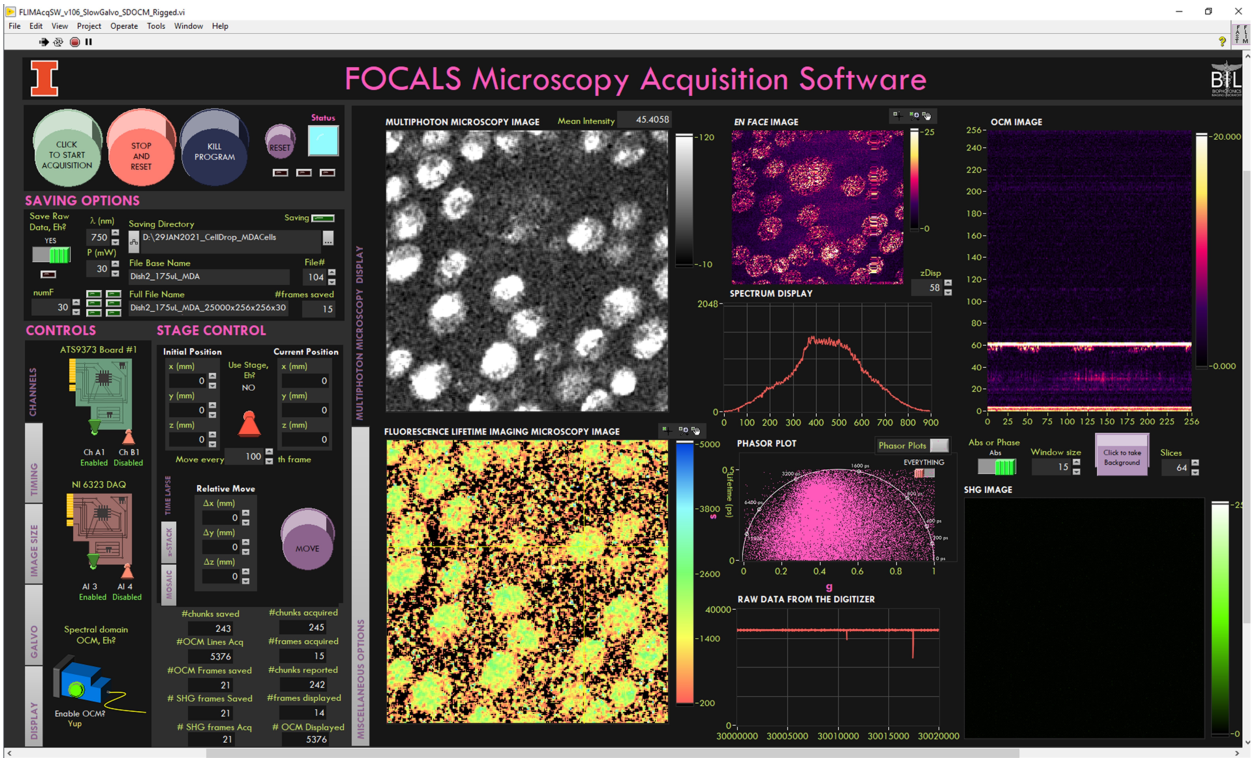

Supplementary Figure 8. **Screenshot of the FOCALS microscopy acquisition software** for MDA-MB-231 cells on a flat surface, imaged using FLIM and OCM. The graphical user interface, control of the DAQ card, and the frame grabber interface are in LabVIEW (National Instruments Corp.) using a state machine diagram. The fast data acquisition control for digitizer and the control for GPU were written in C. OCM and FLIM data were processed on the GPU on two concurrent streams and sent back to the LabVIEW control for display.

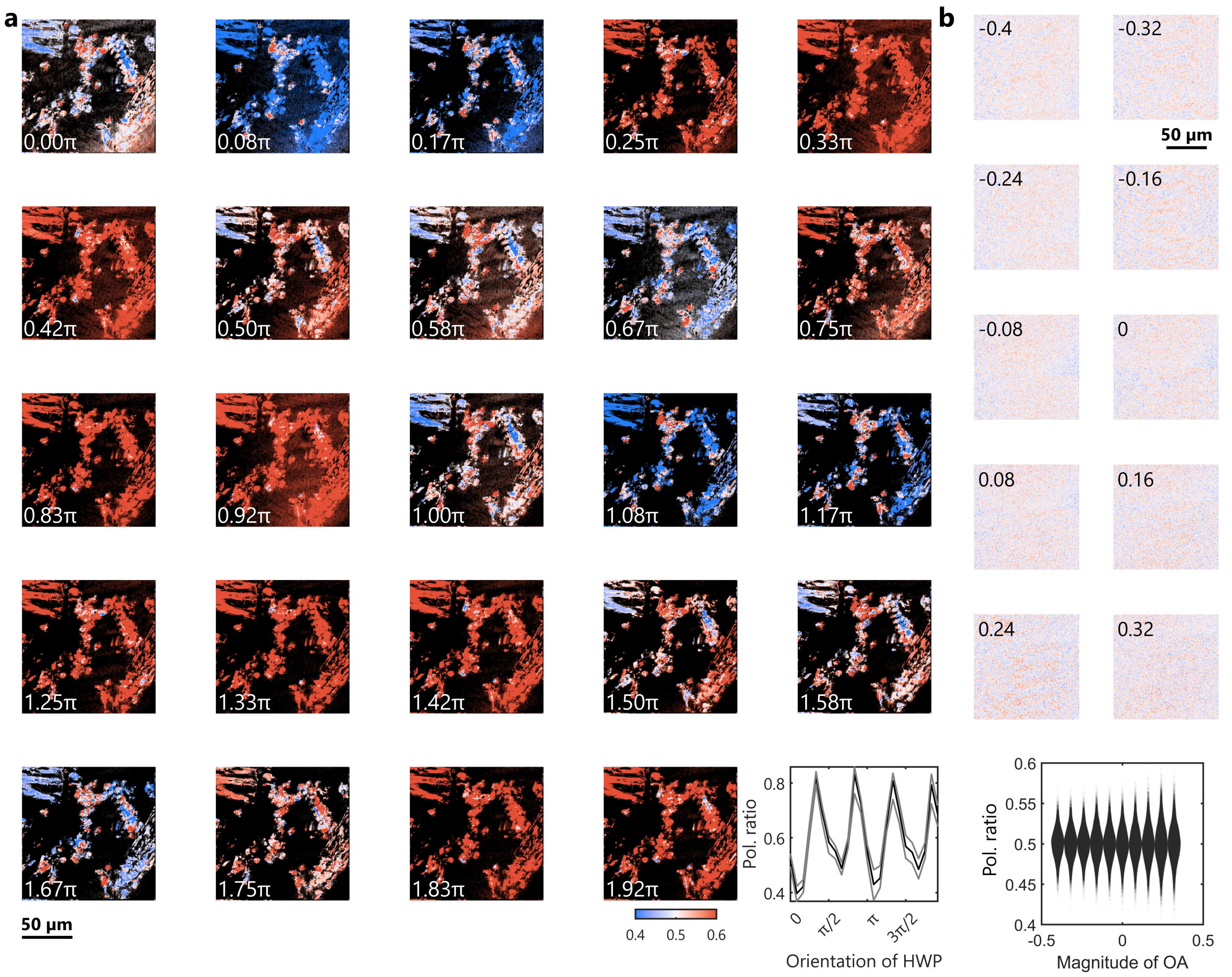

Supplementary Figure 9. **The polarization of PS-OCM in FOCALS microscopy was calibrated by imaging urea-crystals at different incident polarization of the excitation beam**. **a.** Polarization ratio of urea crystals acquired with a half-wave plate placed before the fast galvanometer-scanned mirror. As expected, highly-birefringent urea crystals are highly heterogenous the median ratio follows a sinusoidal pattern when the half-wave plate is rotated at different angles. **b.** Polarization ratio of a glass coverslip with different magnitudes of astigmatism imparted at the DM to show the distribution of the polarization-ratio (Corresponding to Fig. 4a in the main manuscript). The adjacent graphs show the distribution of the polarization ratios for the frames shown.

Supplementary Table 1. Description of the clocks and signals in FOCALS microscopy.

Signal name	Frequency	Duty cycle	Description	Number of clock periods per trigger	Triggered by	Triggers and active edge	Origin	Destination
**Frame clock**	0.4-0.6 Hz	2/256	TTL	∞ (until stopped or enough frames saved)	None	Chunk clock ↑<br>Slow galvo waveform ↑<br>Fast galvo Waveform ↑	DAQ card	Internally routed to DAQ card
**Chunk clock**	$\frac{\text{Line clock rate}}{\frac{\text{numY}}{\text{numChunks}}+\text{idleLines}}$<br>$=\frac{256\text{ Hz}}{\frac{256}{8}+4}=7.11\text{ Hz}$	$\frac{\text{idleLines}}{\text{idleLines}+\frac{\text{numY}}{\text{numChunks}}}$<br>$=\frac{4}{4+\frac{256}{8}}=\frac{4}{36}$	TTL	numChunks	Frame clock ↑	Line clock ↑	DAQ card	Internally routed to DAQ card<br>Buffer trigger of the digitizer
**Fast galvo waveform**	Line clock rate<br>= 256 Hz	0.8	Sawtooth	numY +<br>idleLines •<br>numChunks +<br>numLinesFlyback<br>= 256 + 8 • 4 + 4<br>= 292	Frame clock ↑	-	DAQ card	Galvo driver
**Slow galvo waveform**	Frame rate	-	Progressive ramp<br>(Constant when chunk clock is high, rising when chunk clock is low, flyback at the end)	1	Frame clock ↑	-	DAQ card	Galvo driver
**Line clock**	Line clock rate<br>= 256 Hz	The lag between the galvo signal and positioner feedback	TTL	$\frac{\text{numY}}{\text{numChunks}}$<br>$=32$	Chunk clock ↑	Analog sampling of the FLIM PMT↓<br>A-scan clock ↓<br>Analog sampling of the SHG PMT↓	DAQ card	Line trigger of the digitizer<br>Internally routed to DAQ card
**A-scan clock (Pixel clock)**	$\frac{\text{Laser clock rate}}{\text{num Pulses per Pixel}}$<br>$=\frac{80\text{ MHz}}{625}=128\text{ kHz}$	Exposure time •<br>Pixel clock rate<br>= 0.8576	TTL	numX	Line clock ↓	Acquisition of 1 a-scan from the OCT line-scan camera ↓	DAQ card	Internally routed to DAQ card
**Signal from SHG PMT (+ TIA)**	Sampled at Pixel clock rate = 128 kHz<br>(Bandwidth: 60 MHz)	-	Analog, routed through the 60-MHz TIA	numX	Line clock ↓	-	SHG PMT, routed through the 60-MHz TIA	DAQ card
**10 MHz reference clock**	10 MHz	0.5	TTL	∞ (until the laser is turned off)	-	Synchronization clock (through PLL) to the digitizer and DAQ	Sine-to-TTL converter	Reference clock of DAQ card and digitizer
**Downsampled laser clock**	10 MHz	0.5	Sine	∞ (until the laser is turned off)	-	-	Clock downsampling module	Sine-to-TTL converter
**Laser clock**	80 MHz	0.5	Sine	∞ (until the laser is turned off)	-	-	Laser output sampled using 100-MHz bandwidth photodiode	Clock downsampling module
**Signal from FLIM PMT (+ TIA)**	Bandwidth<br>= 1500 MHz	-	Analog, routed through the 1500-MHz TIA	numX •<br>num Pulses per Pixel •<br>$\frac{\text{FLIM Sample clock rate}}{\text{Laser clock rate}}$<br>$=256\cdot 625\cdot\frac{3.2\text{ GHz}}{80\text{ MHz}}$<br>$=6,400,000$	Line clock ↓	-	FLIM PMT, routed through the 1500-MHz TIA	Digitizer
**Sample clock for FLIM**	3200 MHz	0.5	TTL	256 • 625 • 40<br>= 6,400,000	Line clock ↓	Acquisition of one sample from the digitizer	Generated internally in the digitizer based on the 10 MHz reference clock	Internally routed to the digitizer based on the ref clock

Supplementary Table 2. List of the image and acquisition parameters in Supplementary Table 1.

Parameter	Value
Number of pixels along *x* (numX)	256
Number of pixels along *y* (numY)	256
Number of samples along λ (numK)	896
Number of pixels along *z* (numZ)	256
Number of Chunks (numChunks)	8
Number of idle A-scans between chunks (idleLines)	4
Number of pulses per pixel	625
Number of samples per pulse	40
Laser clock rate	80 MHz
Reference clock	10 MHz

Supplementary Table 3. Sizes of raw and processed data in FOCALS microscopy.

Data	Size
Raw FLIM data<br>(per *xy* frame)	numX • numY •<br>Number of pulses per pixel •<br>Number of Samples per pulse<br>= 1,638,400,000 samples<br>= 3.2768 GB
Raw OCM data<br>(per *xyk* volume)	numX • numY • numK<br>= 58,720,256 samples<br>= 117 MB
Raw (and processed) SHG data<br>(per xy frame)	numX • numY<br>= 65,536 samples<br>= 524 kB
Processed FLIM image<br>(per xy frame)	numX • numY<br>= 65,536 samples<br>= 262 kB
Processed FLIM g and s values<br>(per xy frame)	numX • numY • 2<br>= 65,536• 2 samples<br>= 262 kB
Processed MPM image<br>(per xy frame)	numX • numY<br>= 65,536 samples<br>= 262 kB
Processed OCM<br>(per *xyz* volume)	numX • numY • numZ<br>= 16,777,216 samples<br>= 67 MB

Supplementary Movie 1. FLIM and OCM images of MDA-MB-231 cells dropped onto a glass surface acquired at a rate of 0.4 frames per second to track the arrival and motion of individual cells between successive frames on both imaging modalities (FLIM and OCM). The accompanying plots track the changes to the mean OCM and MPM intensities and the mean NAD(P)H fluorescence lifetime.

Supplementary Movie 2. Screen capture of the FOCALS microscopy acquisition software for imaging NE-4C cells at various fields-of-views and focal planes. Each frame spans 128×128 μm^2 across 256×256 pixels.
